# Explainable medical imaging AI needs human-centered design: guidelines and evidence from a systematic review

**DOI:** 10.1038/s41746-022-00699-2

**Published:** 2022-10-19

**Authors:** Haomin Chen, Catalina Gomez, Chien-Ming Huang, Mathias Unberath

**Affiliations:** Department of Computer Science, Johns Hopkins University, Baltimore, MD USA

**Keywords:** Biomedical engineering, Image processing

## Abstract

Transparency in Machine Learning (ML), often also referred to as interpretability or explainability, attempts to reveal the working mechanisms of complex models. From a human-centered design perspective, transparency is not a property of the ML model but an affordance, i.e., a relationship between algorithm and users. Thus, prototyping and user evaluations are critical to attaining solutions that afford transparency. Following human-centered design principles in highly specialized and high stakes domains, such as medical image analysis, is challenging due to the limited access to end users and the knowledge imbalance between those users and ML designers. To investigate the state of transparent ML in medical image analysis, we conducted a systematic review of the literature from 2012 to 2021 in PubMed, EMBASE, and Compendex databases. We identified 2508 records and 68 articles met the inclusion criteria. Current techniques in transparent ML are dominated by computational feasibility and barely consider end users, e.g. clinical stakeholders. Despite the different roles and knowledge of ML developers and end users, no study reported formative user research to inform the design and development of transparent ML models. Only a few studies validated transparency claims through empirical user evaluations. These shortcomings put contemporary research on transparent ML at risk of being incomprehensible to users, and thus, clinically irrelevant. To alleviate these shortcomings in forthcoming research, we introduce the *INTRPRT guideline*, a design directive for transparent ML systems in medical image analysis. The *INTRPRT guideline* suggests human-centered design principles, recommending formative user research as the first step to understand user needs and domain requirements. Following these guidelines increases the likelihood that the algorithms afford transparency and enable stakeholders to capitalize on the benefits of transparent ML.

## Introduction

There have been considerable research thrusts to develop Machine Learning (ML) models in the healthcare domain that assist clinical stakeholders^1^. However, translating these ML models from the bench to the bedside to support clinical stakeholders during routine care brings substantial challenges, among other reasons, because of the high stakes involved in most decisions that impact human lives. When stakeholders interact with ML tools to reach decisions, they may be persuaded to follow ML’s recommendations that may be incorrect or promote unintended biases against vulnerable populations, all of which can have dreadful consequences^2^. These circumstances motivate the need for trustworthy ML systems in healthcare and have sparked efforts to specify the different requirements that ML algorithms should fulfill. Most of these recent efforts focus on achieving a certain on-task performance requirement but neglect that for assisted decision making not ML system performance alone, but human-ML team performance is the most pertinent to patient outcome. How to achieve adequate human-machine teaming performance, however, is debated. While some argue that rigorous algorithmic validation, e.g., similar to the evaluation of drugs, tests, or devices, demonstrates safe and reliable operation and may thus be sufficient for successful human-machine teaming^3,4^, others reason that transparency in an ML model, e.g., by revealing its working mechanisms and presenting a proper interface, is necessary to invoke user trust and achieve the desired human-machine teaming performance^5–7^. The growing interest and convergence of recent works on the importance and need of transparency have stressed that not addressing the opacity of ML techniques might hinder their adoption of in healthcare, limiting the potential positive impacts^5,8–12^. The inability to make the decision making process transparent might affect the misuse and disuse of ML models in the clinical domain, as the utility of the model might be limited if it does not reveal the reasoning process, limitations, and biases^9^. We believe that this dichotomy is artificial in that, first, rigorous validation and transparency are not mutually exclusive, and second, both approaches augment an ML model with additional information in hopes to justify (in other words, make transparent) the recommendation’s validity which is hypothesized to achieve certain human-factors engineering goals such as understandability, reliability, trust and etc. However, as we will highlight in detail through a systematic review, current approaches that aim at advancing human factors goals of ML systems rely on developers’ intuition rather than considering whether these mechanisms affect users’ experience with the system and their ability to act on ML model’s outputs.

Designing ML algorithms that are transparent is fundamentally different from merely designing ML algorithms. The desire for transparency adds a layer of complexity that is not necessarily computational. Rather, it involves human factors, namely the users to whom the ML algorithm should be transparent. As a consequence, transparency of an algorithm is not a property of the algorithm but a relationship between the transparent ML algorithm and the user processing the information. Such relationship can be understood as an *affordance*, a concept that is commonly employed when designing effective Human-Computer Interactions (HCIs)^13^, and we argue that transparency in ML algorithms should be viewed as such. There are several consequences from this definition:

- Developing transparent ML algorithms is not purely computational.
- Specific design choices on the mechanisms to achieve explanations or interpretations may be suitable for one user group, but not for another.
- Creating transparent ML systems without prior groundwork to establish that it indeed affords transparency may result in misspent effort.

Given the user- and context-dependent nature of transparency, it is essential to understand the target audience and to validate design choices through iterative empirical user studies to ensure that design choices of transparent models are grounded in a deep understanding of the target users and their context. In addition, to maintain a user-centered approach to design from the early stages, rapid prototyping with users provides feedback on the current, low- to high-fidelity embodiment of the system that is going to be built eventually. Involving users early by exposing them to low-fidelity prototypes that mimic final system behavior allows designers to explore multiple alternatives before committing to one pre-determined approach that may not be understandable nor of interest to end users.

However, following a human-centered design approach to build transparent ML systems for highly specialized and high stakes domains, such as healthcare, is challenging. The barriers are diverse and include: (1) the high knowledge mismatch between ML developers and the varied stakeholders in medicine, including providers, administrators, or patients; (2) availability restrictions or ethical concerns that limit accessibility of potential target users for iterated empirical tests in simulated setups for formative research or validation; (3) challenges inherent to clinical problems, including the complex nature of medical data (e.g., unstructured or high dimensional) and decision making tasks from multiple data sources; and last but not least, (4) the lack of ML designers’ training in design thinking and human factors engineering.

Starting from the considerations around designing and validating transparent ML for healthcare presented above, we investigate the current state of transparent ML in medical image analysis, a trailblazing application area for ML in healthcare due to the abundance and structure of data. Through a systematic review based on these aspects, we first identify major shortcomings in the design and validation processes of developing transparent ML models. These deficiencies include the absence of formative user research, the lack of empirical user studies, and in general, the omission of considering ML transparency as contingent on the targeted users and contexts. Together, these shortcomings of contemporary practices in transparent ML development put the resulting solutions at substantial risk of being unintelligible to the target users, and consequently, irrelevant.

**Fig. 1:**
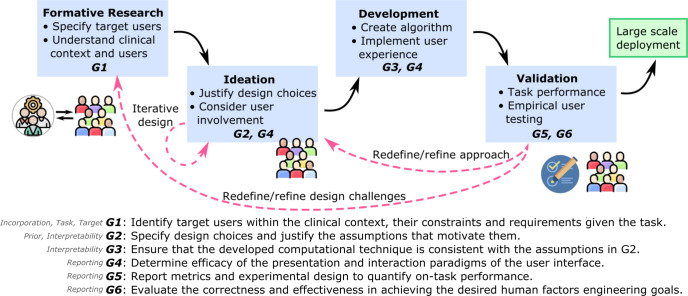
Schematic representation of the *INTRPRT guideline* within the main stages of a human-centered design process. The blue boxes demonstrate the process from understanding end users and their context to the validation of the developed system, which ultimately might result in large scale deployment. The guidelines are summarized below and are located within the design phases based on the aspects pertinent to each one and the corresponding themes of each guideline are listed on the left. Opportunities for iterative design are illustrated with the dashed arrows.

This paper aims to encourage model designers to actively consider and work closely with the end users during the design, construction, and validation of ML models for medical imaging problems. Acknowledging the barriers to widespread adoption of human-centered design techniques to develop transparent ML in healthcare and grounded in our systematic review of the literature, we further propose the *INTRPRT guideline* to help model designers for developing transparent ML for medical image analysis step by step. Figure 1 summarizes our guideline within a human-centered design process. The guideline aims at highlighting the need to ground and justify design choices in a solid understanding of the users and their context when adding transparency or other human factors-based goals to ML systems for medical image analysis. By raising awareness of the user- and context-dependent nature of transparency, designers should consider a trade-off between efforts to (1) better ground their approaches on user needs and domain requirements and (2) commit to technological development and validation of possibly transparent systems. In this way, the guideline may increase the likelihood for algorithms that advance to the technological development stage to afford transparency, because they are well grounded and justified in user and context understanding. This may mitigate misspent efforts in developing complex systems without prior formative user research, and help designers make accurate claims about transparency and other human factors engineering goals when building and validating the model. To the best of our knowledge, we provide the first guidelines for models that afford transparency and involve end users in the design process for medical image analysis.

## An overview of current trends in transparent machine learning development

**Fig. 2:**
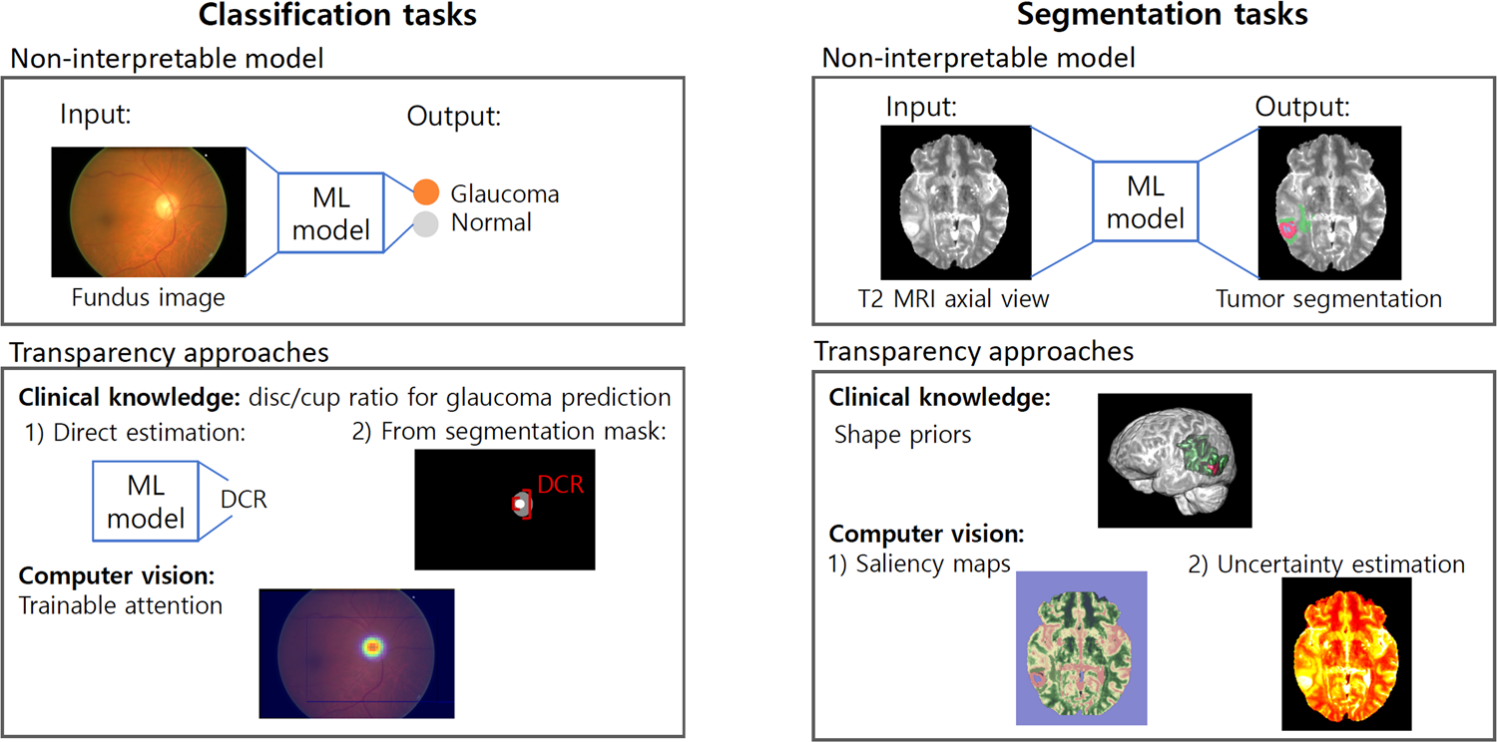
Illustrative examples of different techniques used in transparent ML systems for classification and segmentation tasks from the systematic review. Within each task, a non-interpretable model generates the task outcome from the input image (top). The use of clinical knowledge or computer vision information as priors attempts to add transparency in the outcome generation process (bottom). Images retrieved from the ORIGA^127^ and BraTS2020 datasets^128^.

Compared to developing generic ML algorithms, designing and validating transparent ML algorithms in medical imaging tasks requires consideration of human factors and clinical context. We group these additional considerations into six themes according to the initial review, iteratively defined prior to data extraction and abbreviated to *INTRPRT*; the themes are incorporation (IN), interpretability (IN), target (T), reporting (R), prior (PR), and task (T). *Incorporation* refers to the communication and cooperation between designers and end users before and during the construction of the transparent model. Formative user research is one possible strategy that can help designers to understand end users’ needs and background knowledge^14,15^, but other approaches exist^16^. *Interpretability* considers the technicalities of algorithmic realization of a transparent ML system. Figure 2 provides illustrative examples of some of these techniques. *Target* determines the end users of the transparent ML algorithms. *Reporting* summarizes all aspects pertaining to the validation of transparent algorithms. This includes task performance evaluation as well as the assessment of technical correctness and human factors of the proposed transparency technique (e.g., intelligibility of the model output, trust, or reliability). *Prior* refers to previously published, otherwise public, or empirically established sources of information about target users and their context. This prior evidence can be used to conceptualize and justify design choices around achieving transparency. Finally, *task* specifies the considered medical image analysis task, such as prediction, segmentation, or super resolution, and thus determines the clinical requirements on performance. We emphasize that these themes should not be considered in isolation because they interact with and are relevant to each other. For example, technical feasibility of innovative transparency mechanisms based on the desired task may influence both, the priors that will be considered during development as well as the incorporation of target users to identify and validate alternatives.

Having identified and refined the themes iteratively after an initial review, we structured the systematic review according to the six themes. We identify and summarize dominant trends among the 68 included studies aiming to design transparent ML for medical image analysis. In the *incorporation* theme, cross-disciplinary study teams may constitute a first step towards incorporating target users during ML design, however, only 33 of the included articles were authored by multidisciplinary clinician-engineering teams. More importantly, no paper introduced formative user research to understand user needs and contextual considerations before model construction, which is reflected in the lack of justifying the *prior* theme. Around half of the selected articles (*n* = 28) chose clinical priors and guidelines as an inspiration for transparent systems. In the *target* theme, we found that only 30 of the included articles specified end users, and all of these papers were aimed at clinical care providers, a stark imbalance considering the variety of stakeholders. In the *task* theme, prediction tasks were by far the most common application for transparent ML algorithm design (57/68). In the *Interpretability* theme, methods relying on clinical guidelines resulted in algorithms that adopted multiple sub-steps of a clinical guideline to build the model and generate outcomes, while methods that were based on computer vision techniques for transparency most commonly relied on post-hoc explanations. In the *Reporting* theme, the methods used for assessing transparency varied with the problem formulation and transparency design, and included human perception, qualitative visualizations, quantitative metrics, and empirical user studies; we note that an evaluation with end users was highly uncommon (only 3 of the 68 included studies). However, no paper considered the six themes comprehensively. More importantly, there is no evidence that any papers considered the dependency and interaction between different themes. The reviewed literature further supports that one guideline considering all themes and the interaction between them is highly desired in the medical image analysis community to construct transparent ML models following human-centered design practices.

## INTRPRT guideline

We distilled a set of guidelines for designing transparent ML models according to the interaction and relevancy among the six themes, which is proposed here as *INTRPRT guideline*. The *INTRPRT guideline* provides suggestions for designing and validating transparent ML systems in healthcare in hopes to increase the likelihood that the resulting algorithms indeed afford transparency for the designated end users. The guidelines also address the challenges of following a human-centered design approach in the healthcare domain, propose potential solutions, and apply to different kinds of transparency ML algorithms. To further illustrate the *INTRPRT guideline*, we introduce a case study (see Supplementary information A).

### Guideline 1: specify the clinical scenario, constraints, requirements, and end users

**Fig. 3:**
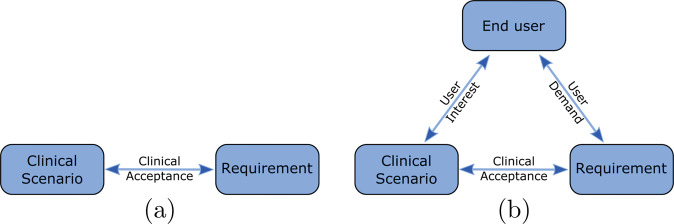
Relationships of components in ML systems. **a** Shows the relationship between the clinical scenario and requirements in non-transparent ML systems, where the system may not be human-facing, and as such, meeting quantitative performance requirements is sufficient. **b** Shows the relationship between the clinical scenario, requirements, and end users in transparent ML systems, as they arise in a human-centered system that seeks to enable users to accomplish a certain task.

The first step to designing any ML algorithm for healthcare is to well define the clinical scenario, the constraints the solution will have to abide by, and all hard or soft requirements the algorithm needs to meet for the clinical *task* to be addressed adequately (cf. Fig. 3). For ML algorithms that do not attempt to be transparent, it is essential but sufficient to assess whether the envisioned ML algorithm design will satisfy the clinical constraints and requirements, e.g., an acceptable classification accuracy in allowable processing time. In addition, when designing transparent ML algorithms it is equally critical to determine and characterize the end users. It is of particular importance to investigate end user characteristics specifically in the clinical context of the chosen task. This is because, depending on the task, stakeholders have varied interest, prior knowledge, responsibilities, and requirements^17^. Deep understanding of the role target users play in the chosen clinical task and their unique needs is critical in determining how to achieve transparency (Guideline 2).

### Guideline 2: justify the choice of transparency and determine the level of evidence

There exists a wide gap in domain expertise and contextualization between target users and ML model designers in most use cases in healthcare. Furthermore, there are multiple “transparency” techniques and choices, such as the transparent working mechanism or user-centered interactions in practice. Simply selecting a “transparency” technique, without incorporating and consulting target users puts the resulting ML models at risk of not achieving the desired transparency. The human-centered design approach addresses this challenge through iterative empirical studies that over time guide the development and refinement of the technical approach such that, upon completion, the design choices are well justified by empirical target user feedback. This approach may not always be feasible in healthcare due to accessibility and availability barriers of target users. To address this limitation while still enabling technological progress in transparent ML, we introduce four distinct levels of evidence. These levels allow designers to classify the level of confidence one may have that the specific design choices will indeed result in a model that affords transparency.

The levels of evidence are based on increasingly thorough approaches to understand the chosen end users in context of the envisioned task:

- Level 0: No evidence. No dedicated investigations about the end users are performed to develop transparent ML systems.
- Level 1: One-way evidence. Formative user research techniques, such as surveys and diary studies, are only performed once without further feedback from end users about the findings extracted from the research phase, resulting in one-way evidence. Such user research suffers risks of potential bias in concluding about justification of transparency because there is no opportunity for dialog, i.e., designers may ask irrelevant questions or target users may provide non-insightful, potentially biased responses.
- Level 2: Public evidence. Public evidence refers to information about target user knowledge, preference, or behavior that is public domain and vetted in a sensible way. Public evidence includes clinical best practice guidelines, Delphi consensus reports, peer-reviewed empirical studies of closely related approaches in large cohorts, or well documented socio-behavioral phenomena.
- Level 3: Iteratively developed evidence. Iteratively developed evidence is transparency evidence that is iteratively refined through user feedback where designers and end users communicate with each other throughout method development. The purpose of iteratively validating and refining the current transparency mechanism is to identify any potential bias in the assumptions that motivate the transparency technique while ensuring that it is understandable to end users.

Being actively cognizant of the level of evidence that supports the development enables trading off development efforts between ML method development vs. gathering richer evidence in support of the intended developments.

### Guideline 3: clarify how the model follows the justification of transparency

This guideline is designed to ensure that the transparency technique used in the ML model is indeed consistent with the assumptions made during its justification. While complying with this guideline is trivial if the model is developed in a human-centered design approach (Level 3: Iteratively developed evidence), in all other cases designers should be explicit about the intellectual proximity of the developed technical approach to the motivating evidence. To this end, after specifying which components of the ML model require transparency for users to capitalize on the intended benefits, it is desirable for the method to be as simple as possible so that it can be easily derived from and linked to the justification of transparency. Once confirmed that the envisioned model is indeed consistent with the justification, computational development of the model, including training, refinement, and validation, begins.

### Guideline 4: determine how to communicate with end users

In addition to content (Guidelines 1, 2, and 3), seemingly peripheral factors on the presentation of information may play a disproportionate role in the perception of transparency. It is well known that factors like format (e.g., text, images, plots)^18^, channel (e.g. graphical interface)^19^, and interactivity (e.g. whether users can provide feedback or refine model outputs) can drastically affect users’ experience and performance^20–22^, and therefore, must be aligned with the goal of transparent system development. Clearly, the selection of presentation mode should be incorporated early and supported by some degree of evidence, that emerges naturally when following human-centered design principles but requires justification if not (as posited for transparency in Guideline 2). Ultimately, users’ experience with the systems plays an important role in their willingness to adopt it in a real setup^21,23^.

### Guideline 5: report task performance of the ML systems

Similar to ordinary ML models, the transparent system must be evaluated quantitatively using appropriate metrics that well reflect the desired performance. In addition, the data used to evaluate the algorithm and its relevance regarding the clinical target task must be specified. Metrics and evaluation protocols should be selected to well determine the model’s abilities in regard to the clinical requirements specified per Guideline 1. Reporting task performance of the algorithm in standalone deployment is important as a baseline for empirical studies in which users may interact and collaborate with the system to complete a task, and team performance (human + ML system) metrics can be measured. Such comparisons are relevant to the goal of improving team performance when integrating intelligent systems to assist humans in complex tasks^23,24^.

### Guideline 6: assess correctness and human factors of system transparency

In addition to task performance, transparent ML systems must be evaluated with respect to their transparency claims. This validation is generally necessary even for Level 3 (iteratively developed evidence) justified transparency mechanisms, because user studies in the design phase commonly rely on mock-up prototypes of the envisioned system, and therefore, may have different modes of failure than the final ML system. Within this guideline, we distinguish two types of evaluation: (1) Validating the correctness of the transparency technique, which objectively assesses whether the information supplied to achieve transparency is in agreement with the justification. Achieving correctness is particularly important for systems that rely on post-hoc explanations, since explanations may rely on a second model that is distinct from the ML algorithm generating recommendations. (2) Validating the effectiveness of transparency in a human-centered approach, to demonstrate that the transparent ML system applied to relevant data samples and in cooperation with target users achieves the desired goals. This empirical evaluation determines the efficacy of human factors engineering. The dimensions that are often considered include users’ trust^18^, reliance, satisfaction^22^, mental model formation^25^, and system acceptance. Reporting of these user studies should include details of the experimental design, participant sample, and techniques to analyze the results.

Transparency of ML algorithms for medical image analysis is commonly motivated by the desire of automating complex tasks while retaining a clear interface for human interaction, e.g., to improve trust, avoid over-reliance, or increase acceptance. However, achieving these design goals through transparency requires the development of transparent ML algorithms that are intelligible by the envisioned end users. In design thinking, aligning technological developments with user needs is accomplished through user involvement in the design process and iterative user testing, which is largely infeasible in healthcare due to varied barriers to end user involvement. We propose a design and evaluation framework where ML designers actively consider end users’ needs, knowledge, and requirements, allowing designers to classify the reliability of their understanding of end user needs using four levels of evidence. Explicitly thinking about the confidence one may have in the assumptions about end users that drive transparent ML system development may mitigate the risks of developing solutions that are unintelligible to the target users, and therefore, neither achieve the desired human factors engineering goals nor benefit clinical practice. Similarly, quantifying the level of evidence currently available to motivate transparency claims then allows developers to trade-off resources between technical ML developments and additional formative research of their target users to ensure that the resulting systems are fit to meet the requirements of the clinical task but foremost the target users.

## Results and discussion

We discuss the *INTRPRT guideline* in the context of the key observations from different themes of our systematic review to identify opportunities to improve the design of transparent ML systems for medical image analysis. Each discussion point focuses on one or more themes that we introduced before. Furthermore, the second last subsection presents successful examples of ML systems designed with clinical end users and the last subsection also includes the comparison of our guideline and systematic review with existing literature.

### Importance of formative user research and empirical user testing

Both formative user research (theme *incorporation* and *prior*) and empirical user testing (theme *reporting*) are critical to ensure that solutions meet user needs (theme *target*). On the one hand, formative user research helps designers navigate and understand end users’ domain practice and needs. On the other hand, empirical user testing assesses whether the designed algorithm indeed achieves the human factors engineering goals, such as affording transparency, promoting trust, or avoiding over-reliance. Additionally, early user involvement in the design process using prototypes of increasing fidelity provides opportunities to review and iterate over design choices. From our systematic review of the literature presented in the “Detailed Analysis of Findings during Systematic Review” section, we find that although most contemporary studies on transparent ML formulate human factor engineering goals, no study reported formative user research or empirical testing to inform and validate design choices. We must conclude that contemporary research efforts in medical image analysis have disproportionately prioritized the technological development of algorithmic solutions that alter or augment the predictions of complex ML systems with the implicit—though unfortunately often explicit—assumption that those changes would achieve transparency. However, because of the substantial knowledge imbalance between ML engineers and target users among other reasons detailed above, it is unlikely that, without formative user research or empirical tests, those systems truly afford transparency or achieve the promised human factors engineering goals. While demonstrating the computational feasibility of advanced transparency techniques is certainly of interest, grounding the need for these techniques in solid understanding of the target users should be the first step for most, if not all, such developments.

### General assessment of transparency

In addition to user involvement or formative research during the design phase (theme *incorporation* and *prior*), upon completion of ML development, system transparency needs to be empirically validated (theme *reporting*). During the literature review, we observed that hardly any study reported quantitative empirical user evaluations as part of final method validation, and many of the included articles limited analysis of transparency goals to qualitative analysis by presenting a limited number of illustrative examples, e.g., pixel-attribution visualizations. While such analysis may suggest fidelity of the transparency design to the cause of the prediction in those few select samples, its utility beyond is unclear. In cases where no empirical user evaluation is conducted, neither during conceptualization nor during development, claims around system transparency or human factors are at high risk of being optimistic and should be avoided.

### Transparent machine learning systems for diverse stakeholders

The purpose of adding transparency to an ML model varies across end users and their context, which we covered in the *target* theme. The current literature on transparent ML for medical image analysis focuses heavily on care providers. In fact, all of the included articles that explicitly specify end users targeted clinicians, such as radiologists, pathologists, and physicians. However, clear opportunities for transparent ML systems exist for other clinical stakeholders, such as other care team members including nurses or techs, healthcare administrators, insurance providers, or patients. Designing transparent ML systems for these stakeholder groups will likely require different approaches, both technological as well in regards to human factors engineering, because these target users are likely to exhibit distinct needs, requirements, prior knowledge, and expectations. In light of recent articles that question the utility of transparency in high stakes clinical decision making tasks^3,26^, driving transparent ML development using a “human factors first” mindset while expanding target user considerations to more diverse stakeholder groups may increase the likelihood of transparent ML having an impact on some aspects of the healthcare system.

### Transparency for tasks with and without human baseline

Clearly specifying and formulating the medical task that the ML system solves is fundamental to determine the assistance that it can provide to clinical practice. Along with the disproportionate consideration of clinicians as end users goes a disproportionate focus on clinical tasks that are routinely performed in current clinical practice (*n* = 60/68) by those target users. One motivation for investigating transparency in such tasks is the existence of clear and systematic clinical workflows and guidelines, e.g., the Breast Imaging Reporting and Data System (BI-RADS) system for mammography, the AO/OTA Tile grading of pelvic fractures, or other easily intelligible covariates associated with outcomes. The availability of such human-defined baselines that are already used for clinical decision making provides immediate Level 2 evidence of transparency for ML systems attempting their replication. In addition, it facilitates data collection and annotation, because intermediate outputs that may be required to build such system are known a priori. Conversely, justifying specific attempts at achieving transparency is much more complicated for tasks that do not readily have human-based baselines or clinical best practice guidelines. Some such tasks may already be performed in clinical practice, such as segmentation or super-resolution, the interpretation of which may be ambiguous and result in high variability among observers^27^. Other tasks may be beyond the current human understanding of the underlying mechanisms that enable ML-based prediction, e.g., ethnicity prediction from chest X-ray^28^ or various tasks in digital pathology^29,30^. In these scenarios, while it may be possible to derive some justification from the literature, e.g., how target users generally approach tasks of the kind, achieving even Level 2 justification is difficult if not impossible. Empirically validating the envisioned mechanisms for transparency with respect to their ability to afford transparency and achieve the human factors engineering goals is thus paramount when attempting to benefit such tasks.

### Successful examples of machine learning systems designed with clinical end users

Early identification and direct communication with end users, as it is emphasized in the *target* and *incorporation* themes, allows ML designers to bridge the knowledge gap and design for users in highly specialized contexts. By following human-centered design and HCI practices, previous works have illustrated ways to incorporate end users in the design process of ML systems for clinical applications. For instance, target users were consulted in the design of an ML tool in an image retrieval system for medical decision making^14^, enabling the team to design a system that preserves human agency to guide the search process. Through an iterative design process, functional prototypes of different refinement techniques based on documented user needs were implemented and further validated in a user study. To enable users to explore and understand an Artificial Intelligence (AI) enabled analysis tool for Chest X-ray (CXR) images, a user-centered iterative design assessed the utility of potential explanatory information in the AI system^15^. Users’ needs during their introduction to an AI-based assistance system for digital pathology were identified through open-ended interviews and a qualitative laboratory study^31^. Iterative co-design processes were followed to identify clinicians’ perceptions of ML tools for real clinical workflows, e.g., antidepressant treatment decisions^16^ and phenotype diagnosis in the intensive care unit^32^. Determining the efficacy of envisioned ML systems or ML-enabled interaction paradigms in empirical user studies before committing resources to their fully-fledged implementation has become common practice in human-centered AI, e.g.,^21,33,34^, with many studies considering tasks that are related to medical image analysis^15,35^. Increasing the acceptance of empirical formative user research as an integral component of human-centered ML design for healthcare tasks, including medical image analysis, will be critical in ensuring that the assumptions on which human-centered systems are built hold in the real world.

### Increasing demand for guidelines to build machine learning systems

Motivated by advances in AI technologies and the wide range of applications in which it can be used to assist humans, there are ongoing efforts to guide the design and evaluation of AI-infused systems that people can interact with (theme *target*). Generally applicable design guidelines were compiled and iteratively refined by HCI experts to design and evaluate human-AI interactions^36^. Although these guidelines are relevant and suitable for a wide range of common AI-enabled systems, more nuanced guidelines are desirable for domains where study participants cannot be recruited nor interviewed in abundance. Similarly, previous attempts to guide the design of effective transparency mechanisms acknowledge that real stakeholders involved should be considered and understood^17,32,37^. Starting from the identification of diverse design goals according to users’ needs and their level of expertise on AI technology, and a categorization of evaluation measures for Explainable Artificial Intelligence (XAI) systems^38^, addressed the multidisciplinary efforts needed to build such systems. A set of guidelines, summarized in a unified framework, suggests iterative design and evaluation loops to account for both algorithmic and human factors of XAI systems. However, similar to ref. ^36^, these guidelines are intended for generic applications, e.g., loan and insurance rate prediction^39^ and personalized advertisements^40^, and do not consider additional challenges, barriers, and limitations when developing algorithms for domains that exhibit users with very specific needs and in highly specific contexts, such as healthcare. Other considerations to build interpretable AI systems have been identified from a multidisciplinary perspective^12^. For instance, the approach presented in ref. ^41^ summarized four guidelines that included the application domain, technical implementation, and human-centered requirements in terms of the capabilities of human understanding. A requirements list formulated as a “fact sheet” was introduced in ref. ^42^ to characterize and assess explainable systems along five key dimensions: functional, operational, usability, safety and validation. While the five dimensions allow to systematically compare and contrast explainability approaches theoretically and practically, the properties that were included failed to consider where and how to formulate the justification of transparency. Formative user research and validation of the justification of transparency are especially essential in healthcare, where a huge knowledge imbalance exists between ML designers and end users of AI systems.

Considering potential uses of AI in clinical setups, there have been efforts to define guidelines for the development and reporting of medical ML systems. For instance, guidelines for clinical trials that involve AI were proposed in ref. ^43^, including items such as the description of intended users, how the AI intervention was integrated, how the AI outputs contributed to decision-making, among others. While specifying these items is also relevant for creating transparent systems, these guidelines do not include requirements in dimensions unique to the transparency of an algorithm, such as its justification and validation. Guidelines for the initial clinical use of AI systems were formulated in ref. ^44^, highlighting the importance to assess the actual impact of an algorithm on its users’ decisions at an early stage. This recommendation of an early and formative evaluation is aligned with our guideline with respect to formative user research during the initial stages to support design choices for transparency. Concerned with the reproducibility and reliability of medical ML studies, a set of practical guidelines as a checklist or questions has been collected for authors and reviews to assess the methodological soundness of contributions^45^, to promote standard reporting practices^46^, and for clinicians to assess algorithm readiness for routine care^47^. Besides the general reporting items regarding the problem definition, data, model, and validation, these checklists consider the definition of the target user and the availability of interpretability information and support for related claims; however, these are questions to be solved once the transparency technique has been incorporated and might lack an appropriate justification and not achieve the intended goals. By considering the reason to demand explainability in advance, which is determined by the application domain and target users of the AI system, model designers can determine the importance and usefulness of the properties offered by certain explainability techniques. To choose among available explainability techniques, a framework with recommendations regarding mostly technical aspects for researchers was proposed in ref. ^8^.

With the trend that ML is more popular in clinical decision making tasks due to its performance, recent surveys and systematic reviews have aimed to summarize existing literature to create transparent ML in healthcare. However, these surveys failed to consider all the themes proposed in this paper and each aspect of transparent ML is reviewed in isolation. More importantly, current reviews mainly focus on the existing transparency techniques and evaluation, ignoring how and where justification of transparency emerges. For example, a survey categorized research works related to the interpretability of ML in general, and then applied the same categories to interpretability in the medical field^48^. In addition to providing an overall perspective of the different interpretable algorithms that are available in the medical field, the survey identified the recurring assumption of having interpretable models without human subject tests, questioning the utility within medical practices and whether ML designs consider actual medical needs. More specifically, there have been surveys focused uniquely on transparent techniques for medical imaging. The interpretability methods to explain deep learning models were categorized in detail based on technical similarities, along with the progress made on the corresponding evaluation approaches in ref. ^9^. Another overview of deep learning-based XAI in medical image analysis is presented in ref. ^49^, considering a variety of techniques that were adapted or developed to generate visual, textual, and example-based explanations in the medical domain. Some of the observed trends and remarks in this survey match our perspective and recommendations in the design of transparent methods for medical imaging, including the lack of evaluation as a standard practice, the user-dependent nature of explanations, and the importance of active collaboration with experts to include domain information. Instead of proposing a general perspective in a broad range of healthcare problems, some reviews focus on specific topics of medical image analysis. Transparent ML for human experts in cancer diagnosis with AI is reviewed in ref. ^10^ with a focus on 2 aspects: ML model characteristics that are important in cancer prediction and treatment; and the application of ML in cancer cases. These two aspects are similar to our proposed theme “Interpretability” and “task”, but we summarize the two themes in the general medical image analysis area instead of limiting to cancer studies, include more on recent studies (starting from 2012), and focus on more recent ML techniques such as Convolution Neural Networks (CNNs). Likewise, transparent ML in cancer detection is also reviewed in ref. ^50^ and structured following the same aspects of generic transparent ML techniques, such as Local vs. Global and Ad-Hoc vs. Post-Hoc. distinctions

The guidelines and systematic review of the state of the field presented here aim at emphasizing the need for formative user research and empirical user studies to firmly establish the validity of assumptions on which human factors engineering goals (including transparency) are based; a natural first step in human-centered AI or HCI, but not yet in medical image analysis. As methods for the human-centered development of transparent ML for medical image analysis mature, the guidelines presented here may require refinements to better reflect the challenges faced then. At the time of writing, supported by the findings of the systematic review, we believe that the lack of explicit formative research is the largest barrier to capitalizing on the benefits of transparent ML in medical image analysis.

To conclude, transparency is an affordance of transparent ML systems, i.e., a relationship between models and end users. Therefore, especially in contexts where there exists a high knowledge gap between ML developers and the envisioned end users, developing transparent ML algorithms without explicitly considering and involving end users may result in products that are unintelligible in the envisioned context and irrelevant in practice. Efforts to build ML systems that afford transparency in the healthcare context should go beyond computational advances, which—based on the findings of our systematic review—is not common practice in the context of transparent ML for medical image analysis. While many of the approaches claimed transparency or derivative accomplishments in human factors engineering, they did so even without defining target users, engaging in formative user research, or reporting rigorous validation. Consequently, for most of the recently proposed algorithms, it remains unclear whether they truly afford transparency or advance human factors engineering goals. We acknowledge that building systems that afford transparency by involving end users in the design process is challenging for medical image analysis and related healthcare tasks. In this context, we propose the *INTRPRT guideline* that emphasize the importance of user and context understanding for transparent ML design, but provide alternatives to empirical studies for formative user research. By following these guidelines, ML designers must actively consider their end users throughout the entire design process. We hope that these design directives will catalyze forthcoming efforts to build transparent ML systems for healthcare that demonstrably achieve the desired human factors engineering goals.

## Methods

### Search strategy and selection criteria

The aim of the systematic review is to survey the current state of transparent ML methods for medical image analysis. Because ML transparency as major research thrust has emerged following the omnipresence of highly complex ML models, such as deep CNNs, we limited our analysis to records that appeared after January 2012, which pre-dates the onset of the ongoing surge of interest in learning-based image processing^51^.

We conducted a systematic literature review in accordance with the Preferred Reporting Items for Systematic reviews and Meta-Analyses (PRISMA) method^52^. We searched PubMed, EMBASE, and Compendex databases to find articles pertinent to transparent ML (including but not limited to explainable and interpretable ML) for medical imaging by screening titles, abstracts, and keywords of all available records from January 2012 through July 2021. Details of the search terms and strategy can be found in Supplementary information B.

### Study selection

**Fig. 4:**
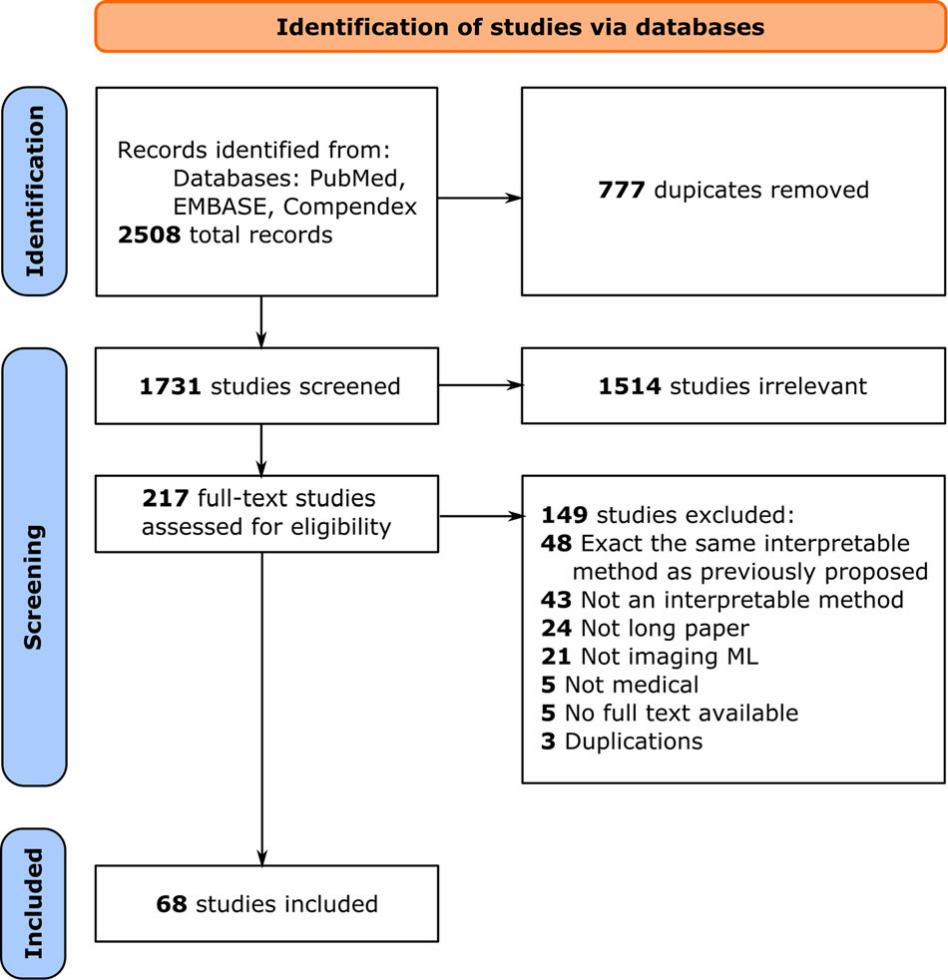
PRISMA diagram for transparent ML in medical imaging. The flow diagram shows the number of records identified, of studies excluded and the reasons for exclusion, and of studies included in our systematic review.

Following the removal of duplicates (1731 remained), studies were first pre-screened using the title and abstract. Studies that did not describe transparent methods nor medical imaging problems were immediately excluded (217 remained). We then proceeded to full-text review, where each study was examined to determine whether the study presented and evaluated a transparent ML technique for medical image analysis. Failure to comply with the described inclusion/exclusion criteria resulted in the study’s removal from further consideration. Detailed statistics and a complete description of the pre-screening and full-text review can be found in Supplementary information C and Fig. 4. 68 articles were included for information extraction.

### Data extraction strategy

For the 68 selected articles that met the inclusion criteria, two authors (H.C. and C.G.) performed detailed data extraction to summarize important information related to the six themes described in INTRPRT Guideline Section. A data extraction template was developed by all authors and is summarized in Supplementary information D. Every one of the 68 articles was analyzed and coded by both authors independently and one author (H.C.) merged the individual reports into a final consensus document. Despite our efforts to broadly cover all relevant search terms regarding transparent ML in medical imaging, we acknowledge that the list may not be exhaustive. There are vast numbers of articles that have imbued transparency in their methodology, but transparency (or contemporary synonyms thereof, such as explainability or interpretability) is not explicitly mentioned in the title, abstract, or keywords of these articles, and often not even in the body of the text^53^. This fact makes intractable to identify all articles about transparent ML methods. Finally, the review is limited to published manuscripts, long articles and novel approaches. Publication bias may have resulted in the exclusion of works relevant to this review.

## Detailed analysis of findings during systematic review

The data extraction template for studies included in the systematic review was structured using the six themes of the *INTRPRT guideline*, the adequacy of which was confirmed during data extraction. Therefore, we summarize our findings for each theme and provide details of the extraction results for each article in Tables 2&3 in Supplementary information D.

### IN: incorporation

A common trend among included studies (*n* = 33) was that the presented methods were developed by multidisciplinary clinician-engineering teams, as was evidenced by the incorporation of clinical specialists, such as physicians, radiologists, or pathologists, in the study team and on the author lists. In light of the current bias towards clinicians as end users of transparent ML algorithms, this observation suggests that designers may have communicated with a limited subset of the intended end users. However, no formative user research is explicitly described or introduced in these articles to systematically understand the end users before implementing the model. Further, we found that incorporating clinical experts did not have a considerable impact on whether clinical priors or standard or care guidelines (i.e., Level 2 evidence) were used to build the ML system (39%/44% articles with/without the incorporation of end users use clinical priors). Regarding the technical approach to provide transparency, the incorporation of medical experts motivated designers to incorporate prior knowledge directly into the model structure and/or inference for medical imaging (73%/64% articles with/without the incorporation of end users do not need a second model to generate transparency).

### IN: interpretability

Transparency of ML systems was achieved through various techniques, including attention mechanisms (*n* = 15), use of human-understandable features (*n* = 11), a combination of deep neural networks and transparent traditional ML methods (*n* = 7), visualization approaches (*n* = 5), clustering methods (*n* = 4), uncertainty estimation/confidence calibration (*n* = 3), relation analysis between outputs and hand-crafted features (*n* = 3), and other custom techniques (*n* = 20).

The use of an attention mechanism was the most common technique for adding transparency. Attention mechanisms enabled the generation of pixel-attribution methods^54^ to visualize pixel-level importance for a specific class of interest^55–69^. In segmentation tasks, where clinically relevant abnormalities and organs are usually of small sizes, features from different resolution levels were aggregated to compute attention and generate more accurate outcomes, as demonstrated in multiple applications, *e.g*., multi-class segmentation in fetal Magnetic Resonance Imagings (MRIs)^58^ and multiple sclerosis segmentation in MRIs^61^. Clinical prior knowledge was also inserted into the attention mechanism to make the whole system more transparent. For instance,^65^ split brain MRIs into 96 clinically important regions and used a genetic algorithm to calculate the importance of each region to evaluate Alzheimer’s Disease (AD).

Human-understandable features, e.g., hand-crafted low-dimensional features or clinical variables (age, gender, etc.) were frequently used to establish transparent systems. There existed two main ways to use human-understandable features in medical imaging: (1) Extracting hand-crafted features, *e.g*., morphological and radiomic features, from predicted segmentation masks generated by a non-transparent model^70–79^ followed by analysis of those hand-crafted features using a separate classification module; (2) Directly predicting human-understandable features together with the main classification and detection task^80–84^. In these approaches, all tasks usually shared the same network architecture and parameter weights.

Instead of explicitly extracting or predicting human-understandable features, other articles further analyzed deep encoded features with human-understandable techniques by following clinical knowledge. Techniques such as decision trees were constructed based on clinical taxonomy for hierarchical learning^76,85–90^. Rule-based algorithms^91^ and regression methods^92^ were used to promote transparency of the prediction^93^ created a Graphical Convolution Network (GCN) based on clinical knowledge to model the correlations among colposcopic images captured around five key time slots during a visual examination.

We also identified various other methods to create transparent systems. These methods can be categorized as visualization-based, feature-based, region importance-based, and architecture modification-based methods. Each approach is discussed in detail below.

Visualization-based methods provide easy-to-understand illustrations by overlaying the original images with additional visual layouts generated from transparency techniques. There existed two main visualization-based methods: (1) Visualizing pixel-attribution maps: These maps may be generated using gradient-based importance analysis^94,95^, pixel-level predicted probability^96^, or a combination of different levels of feature maps^97,98^. (2) Latent feature evolution: Encoded features were evolved according to the gradient ascent direction so that the decoded image (e.g., generated with an auto-encoder technique^99^) gradually change from one class to another^100,101^.

Feature-based methods directly analyze encoded features in an attempt to make the models transparent. Various feature-based transparency method were proposed for transparent learning^102–104^ first encoded images to deep features and then clustered samples based on these deep features for prediction or image grouping tasks. Feature importance was also well-studied to identify features that are most relevant for a specific class by feature perturbation^105,106^ and gradients^107^^,108^ identified and removed features with less importance for final prediction through feature ranking.

As an alternative to measure feature contribution, input region importance was also analyzed to reveal sub-region relevance to each prediction class. Image occlusion with blank sub-regions^109–111^ and healthy-looking sub-regions^112^ was used to find the most informative and relevant sub-regions for classification and detection tasks.

Other approaches modified the network architecture according to relevant clinical knowledge to make the whole system transparent^58^ pruned the architecture according to the degree of scale invariance at each layer in the network^113^ created ten branches with shared weights for ten ultrasound images to mimic the clinical workflow of liver fibrosis stage prediction^114^ aggregated information from all three views of mammograms and used traditional methods to detect nipple and muscle direction, which was followed by a grid alignment according to the nipple and muscle direction for left and right breasts^115^ proposed to learn representations of the underlying anatomy with a convolutional auto-encoder by mapping the predicted and ground truth segmentation maps to a low dimensional representation to regularize the training objective of the segmentation network.

Some other methods used the training image distribution to achieve transparency in classification^116^ used similar-looking images (nearest training images in feature space) to classify testing images with majority votes. Causal inference with plug-in clinical prior knowledge also introduced transparency directly to automatic systems^117–119^. Confidence calibration and uncertainty estimation methods were also used to generate additional confidence information for end users^120–122^.

### T: targets

A striking observation was that none of the selected articles aimed at building transparent systems for users other than care providers. Less than half of the articles explicitly specified clinicians as the intended end users of the system (*n* = 30). From the remaining 38 articles, 17 articles implied that the envisioned end users would be clinicians, while the remaining 21 did not specify the envisioned target users. Articles that were more explicit about their end users were more likely to rely on clinical prior knowledge (Level 2 evidence) in model design. In total, 47% of articles that specified or implied clinicians as end users implemented clinical prior knowledge in the transparent systems while only 18% of articles without end user information use clinical prior knowledge.

### R: reporting

Evaluating different properties of a transparent algorithm besides task-related metrics, especially its performance in regards to achieving the desired human factors engineering goals, complements the assessment of the ML model’s intended purpose. We identified that the quality of the transparency component is currently being evaluated through four main approaches. The first one involves metrics based on human perception, such as the mean opinion score introduced in ref. ^115^ to capture two expert participants’ rating of the model’s outcome quality and similarity to the ground truth on a 5-point scale. Using two study participants, pathologists’ feedback was also requested in ref. ^107^ to assess their agreement with patch-based visualizations that display features relevant for normal and abnormal tissue. The level of agreement was not formally quantified, but reported as a qualitative description. Similarly, one study participant was involved in a qualitative assessment of explanations quality in ref. ^83,108^. These evaluations are different from empirical user studies as they are limited to a few individuals and were mostly used to subjectively confirm the correctness of the transparent component.

The second approach attempted to quantify the quality of explanations for a specific purpose (functionally-grounded evaluation^123^). For instance, some articles evaluated the localization ability of post-hoc explanations by defining an auxiliary task, such as detection^57,88^ or segmentation^62,85,98,112^ of anatomical structures related to the main task. They then contrasted relevant regions identified by the model with ground truth annotations. These quantitative measures (dice score, precision, recall) allowed for further comparisons with traditional explanations methods. Similarly^116^, defined a multi-task learning framework for image classification and retrieval, evaluating retrieval precision and providing a confidence score based on the retrieved neighbors as an attempt to check the learned embedding space. Capturing relevant features consistent with human intuition was proposed in ref. ^106^ by measuring the fraction of reference features recovered, which were defined according to a guideline. Overall, the evaluation of explanations through auxiliary tasks required additional manual efforts to get the necessary ground truth annotations.

Properties of the explanation itself were also quantified as their usefulness to identify risky and safe predictions at a voxel-level for the main task by thresholding on their predictive uncertainty values^122^. Other properties of explanations, such as their correctness (accuracy of rules), completeness (fraction of the training set covered) and compactness (size in bytes) were measured in ref. ^87^. A measure related to completeness was defined in ref. ^88^ and aimed to capture the proportion of training images represented by the learned visual concepts, in addition to two other metrics: the inter- and intra-class diversity and the faithfulness of explanations computed by perturbing relevant patches and measuring the drop in classification confidence. Other articles followed a similar approach to validate relevant pixels or features identified with a transparent method; for example, in ref. ^64^ a deletion curve was constructed by plotting the dice score vs. the percentage of pixels removed and ref. ^55^ defined a recall rate when the model proposes certain number of informative channels^95^ proposed to evaluate the consistency of visualization results and the outputs of a CNN by computing the *L1* error between predicted class scores and explanation pixel-attribution maps. In summary, while the methods grouped in this theme are capable of evaluating how well a method aligns with it’s intended mechanism of transparency, they fall short of capturing any human factors-related aspects of transparency design.

The third, and most common approach, involved a qualitative validation of the transparent systems (*n* = 40) by showing pixel-attribution visualizations overlaid with the input image or rankings of feature relevance, along with narrative observations on how these visualizations may relate to the main task. These qualitative narratives might include comparisons with other visualization techniques in terms of the highlighted regions or the granularity/level of details. Furthermore, following a retrospective analysis, the consistency between the identified relevant areas/features and prior clinical knowledge in a specific task was a common discussion item in 37% of all the articles (*n* = 25); refer to articles^65,86,89,110,117^ for examples. While grounding of feature visualizations in the relevant clinical task is a commendable effort, the methods to generate the overlaid information have been criticized in regards to their fidelity and specificity^53,124^. Further, as was the case for methods that evaluate the fidelity of transparency information, these methods do not inherently account for human factors.

Lastly, transparent systems can be directly evaluated through user studies on the target population, in which the end users interact with the developed ML system to complete a task based on a specific context. In ref. ^96^, the evaluation was centered on the utility of example-based and feature-based explanations for radiologists (8 study participants) to understand the AI decision process. Users’ understanding was evaluated as the accuracy to predict the AI’s diagnosis for a target image and a binary judgment on whether they certify the AI for similar images (and justify using multiple-choice options). Users’ agreement with the AI’s predictions was measured as well. The empirical evidence suggested that explanations enabled radiologists to develop appropriate trust by making an accurate prediction and judgment of the AI’s recommendations. Even though radiologists could complete the task by themselves, a comparison with the team performance was not included, nor the performance of the AI model in standalone operation. An alternative evaluation of example-based explanation usefulness was performed in ref. ^121^, in which pathologists (14 study participants) determined the acceptability of a decision support tool by rating adjectives related to their perceived objectivity, details, reliability, and quality of the system. Compared to a CNN without explanations, the subjective ratings were more positive towards the explainable systems. However, neither the team (expert + AI) nor expert baseline performance was evaluated. The benefit of involving a dermatologist to complete an image grouping task was demonstrated in ref. ^102^, in which domain knowledge was used to constrain updates of the algorithm’s training, resulting in a better grouping performance than a fully automated method. The user evaluation only measured the task performance. These studies that explicitly involve target users to identify whether the envisioned human factors engineering goals were met stand out from the large body of work that did not consider empirical user tests. It is, however, noteworthy that even these exemplary studies are based on very small sample sizes that may not be sufficiently representative of the target users. Careful planning of the study design (including hypothesis statement, experimental design and procedure, participants, and measures) that allows to properly evaluate whether the system achieves the intended goals by adding transparency to the ML system is fundamental, especially considering the resources needed and challenges involved in conducting user testing in the healthcare domain.

Even though there were articles that assessed human factors-related properties of the transparency mechanism, a striking majority of articles did not report metrics beyond performance in the main task (*n* = 49) or did not discuss the transparency component at all (*n* = 9). Task performance was evaluated in the majority of the articles, 91% (*n* = 62), and most of them contrasted the performance of the transparent systems with a non-transparent baseline (*n* = 41). Of those, 36 works (88%) reported improved performance and 5 (12%) comparable results.

### PR: priors

We differentiate two types of priors that can be used as a source of inspiration to devise transparent ML techniques: (1) Priors based on documented knowledge, and especially clinical guidelines considering the unvaried end user specification identified above; and (2) Priors based on computer vision concepts. Most (93%) articles that incorporated clinical knowledge priors (*n* = 28) directly implemented these priors into the model structure and/or inference, while only 68% articles with computer vision priors (*n* = 40) provided transparency by the model itself and/or the inference procedure.

A direct way to include clinical knowledge priors was through the prediction, extraction, or use of human-understandable features. Morphological features, *e.g*., texture, shape and edge features were frequently considered and used to support the transparency of ML systems^70,72,73,75,76,81,83,93^. Biomarkers for specific problems, e.g., end-diastolic volume (EDV) in cardiac MRI^78,79^ and mean diameter, consistency, and margin of pulmonary nodules^80^ were commonly computed to establish transparency. For problems with a well-established image reporting and diagnosis systems, routinely-used clinical features, e.g., Liver Imaging Reporting and Data System (LI-RADS) features for Hepatocellular carcinoma (HCC) classification^84^ or BI-RADS for breast mass^82^ suggested that the ML systems may be intuitively interpretable to experts that are already familiar with these guidelines. Human-understandable features relevant to the task domain were extracted from pathology images, e.g., area and tissue structure features^70^. Radiomic features were also computed to establish the transparency of ML systems^75,125^.

Besides human-understandable features, clinical knowledge can be used to guide the incorporation of transparency within a model. Some articles (*n* = 11) mimicked or started from clinical guidelines and workflows to construct the ML systems^65,74,81,82,106,113–115,118,119,74,113,114^ followed the clinical workflow to encode multiple sources of images and fused the encoded information for the final prediction. Other works followed the specific clinical guidelines of the problems to create transparent systems^65^ split brain MRIs into 96 clinical meaningful regions as would be done in established clinical workflows and analyze all the regions separately. Some other clinical knowledge priors were also presented^85,86,90,126^ established a hierarchical label structure according to clinical taxonomy for image classification^71^ leveraged the transparency from the correlation between the changes of polarization characteristics and the pathological development of cervical precancerous lesions. Clinical knowledge from human experts was used to refine an image grouping algorithm through an interactive mechanism in which experts iteratively provided inputs to the model^102^.

Priors that were derived from computer vision concepts rather than the clinical workflow were usually not specific or limited to a single application. The justification of transparency with computer vision priors was more general than that with clinical knowledge priors. Image visualization-based techniques to achieve transparency were most commonly considered in image classification problems. Common ways of retrieving relevance information were: Visual relevancy through attention^55–64,66–68^; region occlusion by blank areas^109,111^ or healthy-looking regions^112^; and other techniques such as supervision of activated image regions by clinically relevant areas^88,89,92,94,95,97,98^, and image similarity^96^. Feature-based computer vision transparency priors focused on the impact of feature evolution or perturbation on the decoded output. Encoded features were evolved according to the gradient ascent direction to create the evolution of the decoded image from one class to the other^87,100,101^. Some articles directly analyzed the feature sensitivity to the final prediction by feature perturbation^101,105,110^ and importance analysis^77,107,108^, feature distribution^104,105^ or image distribution based on encoded features^103,116^. Confidence calibration and uncertainty estimation also increased the transparency of the ML systems^120–122^.

Even though we attempted to identify the type of prior evidence used to justify the development of a specific algorithm in each ML system, none of the included articles formally described the process to formulate such priors to achieve transparency in the proposed system. While the use of clinical guidelines and routine workflows may provide Level 2 evidence in support of the method affording transparency if the end users are matched with those priors, relying solely on computer vision techniques may not provide the same level of justification. This is because computer vision algorithms are often developed as an analysis tool for ML developers to verify model correctness, but are not primarily designed nor evaluated for use in end user-centered interfaces. The lack of justification and formal processes to inform design choices at the early stages of model development results in substantial risk of creating transparent systems that rely on inaccurate, unintelligible, or irrelevant insights for end users. Being explicit about the assumptions and evidence available in support of the envisioned transparent ML system is paramount to build fewer but better-justified transparent ML systems that are more likely to live up to expectations in final user testing, the resources for which are heavily constrained.

### T: task

Various types of medical image analysis tasks were explored in the included articles. Most of the articles (*n* = 57) proposed transparent ML algorithms for classification and detection problems, such as image classification and abnormality detection. Three-dimensional (3D) radiology images (*n* = 24) and pathological images (*n* = 15) were the most popular modalities involved in the development of transparent algorithms. The complex nature of both 3D imaging in radiology and pathological images makes image analysis tasks more time consuming than 2D image analysis that is more prevalent in other specialities, such as dermatology, which motivates transparency as an alternative to complete human image analysis to save time while retaining trustworthiness. In detail, classification problems in 3D radiological images and pathological images included abnormality detection in computed tomography (CT) scans^56,59,66,73,75,90,95,106^, MRIs^58,60,65,77–79,83,84,98,100,105,110,112,117^, pathology images^55,57–59,62,69–71,77,104,107–109,116,121^ and positron emission tomography (PET) images^63^. Mammography dominated the 2D radiology image applications^76,81,82,87,92,94,114,119,125^, mainly focusing on breast cancer classification and mass detection. For other 2D radiology image applications^96,118^, aimed at pneumonia and pneumothorax prediction from chest X-rays and^113^ created a transparent model for liver fibrosis stage prediction in liver ultrasound images. Classification and detection tasks were explored in other clinical specialities, including melanoma^85^ and skin lesion grade prediction^58,86,87^ in dermatology, glaucoma detection from fundus images^68,74,97^ and retinopathy diagnosis^111^ in ophthalmology, and polyp classification from colonoscopy images in gastroenterology^88,120^.

Segmentation was another major application field (*n* = 9). Research about transparency mainly focused on segmentation problems for brain and cardiac MRIs^61,64,67,72,89,103,115^. Other segmentation problems included mass segmentation in mammograms^76^, cardiac segmentation in ultrasound^115^, liver tumor segmentation in hepatic CT images, and skin lesion segmentation in dermatological images^58^. There also existed other applications, e.g., image grouping in dermatological images^102^ and image enhancement (super resolution task) in brain MRIs^122^ and cardiac MRIs^115^.

Most of the application tasks were routinely performed by human experts in current clinical practice (*n* = 60). A much smaller sample of articles (*n* = 4) aimed to build transparent systems for much more difficult tasks where no human baseline exists, e.g., 5-class molecular phenotype classification from Whole Slide Images (WSIs)^70,88^, 5-class polyp classification from colonoscopy images^120^, cardiac resynchronization therapy response prediction from cardiac MRIs^83^, and super resolution of brain MRIs^122^. The remaining articles (*n* = 4) did not include explicit information on whether human baselines and established criteria exist for the envisioned application, e.g., magnification level and nuclei area prediction in breast cancer histology images^58^, age estimation in brain MRIs^60^, AD status in Diffusion Tensor Images (DTIs), and risk of sudden cardiac death prediction in cardiac MRIs^79^. As previously mentioned, tasks that are routinely performed in clinical evidence may have robust human baselines and clinical guidelines to guide transparent ML development. Applications that are beyond the current possibilities, however, require a more nuanced and human-centered approach that should involve the target end users as early as possible to verify that the assumptions that drive transparency are valid.

## Supplementary information

Supplementary Information of the main article

## Data Availability

Figure 2 contains images from the ORIGA^127^ and BraTS2020 datasets^128^. The ORIGA dataset is a public dataset at Kaggle website (https://www.kaggle.com/datasets/sshikamaru/glaucoma-detection/metadata). The BraTS2020 dataset is also a public dataset at Kaggle website (https://www.kaggle.com/datasets/awsaf49/brats2020-training-data). The authors declare that all the data included in this study are available within the paper and its Supplementary Information files. Please contact author HC to request the data.

## References

[1] Topol EJ (2019). High-performance medicine: the convergence of human and artificial intelligence. Nat. Med..

[2] Obermeyer Z, Powers B, Vogeli C, Mullainathan S (2019). Dissecting racial bias in an algorithm used to manage the health of populations. Science.

[3] Ghassemi M, Oakden-Rayner L, Beam AL (2021). The false hope of current approaches to explainable artificial intelligence in health care. Lancet Digital Health.

[4] McCoy LG, Brenna CT, Chen SS, Vold K, Das S (2022). Believing in black boxes: Machine learning for healthcare does not need explainability to be evidence-based. J. Clin. Epidemiol..

[5] Vellido A (2020). The importance of interpretability and visualization in machine learning for applications in medicine and health care. Neural Comput. Appl..

[6] Char DS, Abràmoff MD, Feudtner C (2020). Identifying ethical considerations for machine learning healthcare applications. Am. J. Bioethics.

[7] Holzinger A, Langs G, Denk H, Zatloukal K, Müller H (2019). Causability and explainability of artificial intelligence in medicine. Wiley Interdiscip. Rev.: Data Mining Knowl. Discov..

[8] Markus AF, Kors JA, Rijnbeek PR (2021). The role of explainability in creating trustworthy artificial intelligence for health care: a comprehensive survey of the terminology, design choices, and evaluation strategies. J. Biomed. Inf..

[9] Salahuddin Z, Woodruff HC, Chatterjee A, Lambin P (2022). Transparency of deep neural networks for medical image analysis: A review of interpretability methods. Comput. Biology Med..

[10] Banegas-Luna AJ (2021). Towards the interpretability of machine learning predictions for medical applications targeting personalised therapies: A cancer case survey. Int. J. Mol. Sci..

[11] Ploug T, Holm S (2020). The four dimensions of contestable ai diagnostics-a patient-centric approach to explainable ai. Artif. Intell. Med..

[12] Amann J, Blasimme A, Vayena E, Frey D, Madai VI (2020). Explainability for artificial intelligence in healthcare: a multidisciplinary perspective. BMC Med. Inf. Dec. Making.

[13] Norman DA (1999). Affordance, conventions, and design. Interactions.

[14] Cai, C. J. et al. Human-centered tools for coping with imperfect algorithms during medical decision-making. In *Proceedings of the 2019 CHI Conference on Human Factors in Computing Systems*, 1–14 (2019).

[15] Xie, Y., Chen, M., Kao, D., Gao, G. & Chen, X. Chexplain: Enabling physicians to explore and understand data-driven, AI-enabled medical imaging analysis. In *Proceedings of the 2020 CHI Conference on Human Factors in Computing Systems*, 1–13 (2020).

[16] Jacobs, M. et al. Designing AI for trust and collaboration in time-constrained medical decisions: A sociotechnical lens. In *Proceedings of the 2021 CHI Conference on Human Factors in Computing Systems*, 1–14 (2021).

[17] Suresh, H., Gomez, S. R., Nam, K. K. & Satyanarayan, A. Beyond expertise and roles: A framework to characterize the stakeholders of interpretable machine learning and their needs. In *Proceedings of the 2021 CHI Conference on Human Factors in Computing Systems*, 1–16 (2021).

[18] Lai, V. & Tan, C. On human predictions with explanations and predictions of machine learning models: A case study on deception detection. In *Proceedings of the conference on fairness, accountability, and transparency*, 29–38 (2019).

[19] Eiband, M. et al. Bringing transparency design into practice. In *23rd international conference on intelligent user interfaces*, 211–223 (2018).

[20] Wang, X. & Yin, M. Are explanations helpful? a comparative study of the effects of explanations in AI-assisted decision-making. In *26th International Conference on Intelligent User Interfaces*, 318–328 (2021).

[21] Cheng, H.-F. et al. Explaining decision-making algorithms through ui: Strategies to help non-expert stakeholders. In *Proceedings of the 2019 chi conference on human factors in computing systems*, 1–12 (2019).

[22] Smith-Renner, A. et al. No explainability without accountability: An empirical study of explanations and feedback in interactive ml. In *Proceedings of the 2020 CHI Conference on Human Factors in Computing Systems*, 1–13 (2020).

[23] Bansal, G. et al. Does the whole exceed its parts? the effect of ai explanations on complementary team performance. In *Proceedings of the 2021 CHI Conference on Human Factors in Computing Systems*, 1–16 (2021).

[24] Bansal G (2019). Beyond accuracy: The role of mental models in human-AI team performance. Proceedings of the AAAI Conference on Human Computation and Crowdsourcing.

[25] Nourani, M. et al. Anchoring bias affects mental model formation and user reliance in explainable ai systems. In *26th International Conference on Intelligent User Interfaces*, 340–350 (2021).

[26] McCoy LG, Brenna CT, Chen S, Vold K, Das S (2021). Believing in black boxes: Machine learning for healthcare does not need explainability to be evidence-based. J. Clin. Epidemiol.

[27] Deeley M (2013). Segmentation editing improves efficiency while reducing inter-expert variation and maintaining accuracy for normal brain tissues in the presence of space-occupying lesions. Phys. Med. Biol..

[28] Banerjee, I. et al. Reading race: Ai recognises patient’s racial identity in medical images. preprint at https://arxiv.org/abs/2107.10356 (2021).

[29] Liu TA (2020). Gene expression profile prediction in uveal melanoma using deep learning: A pilot study for the development of an alternative survival prediction tool. Ophthalmol. Retina.

[30] Lu, M. Y. et al. Deep learning-based computational pathology predicts origins for cancers of unknown primary. preprint at https://arxiv.org/abs/2006.13932 (2020).

[31] Cai CJ, Winter S, Steiner D, Wilcox L, Terry M (2019). "hello AI": Uncovering the onboarding needs of medical practitioners for human-ai collaborative decision-making. Proce. ACM Human-Comput. Interaction.

[32] Wang, D., Yang, Q., Abdul, A. & Lim, B. Y. Designing theory-driven user-centric explainable AI. In *Proceedings of the 2019 CHI conference on human factors in computing systems*, 1–15 (2019).

[33] Nourani, M., King, J. & Ragan, E. The role of domain expertise in user trust and the impact of first impressions with intelligent systems. In *Proceedings of the AAAI Conference on Human Computation and Crowdsourcing*, vol. 8, 112–121 (2020).

[34] Buçinca Z, Malaya MB, Gajos KZ (2021). To trust or to think: Cognitive forcing functions can reduce overreliance on ai in ai-assisted decision-making. Proceedings of the ACM on Human-Computer Interaction.

[35] Gaube S (2021). Do as ai say: susceptibility in deployment of clinical decision-aids. NPJ Digital Medicine.

[36] Amershi, S. et al. Guidelines for human-AI interaction. In *Proceedings of the 2019 chi conference on human factors in computing systems*, 1–13 (2019).

[37] Liao, Q. V., Gruen, D. & Miller, S. Questioning the AI: informing design practices for explainable ai user experiences. In *Proceedings of the 2020 CHI Conference on Human Factors in Computing Systems*, 1–15 (2020).

[38] Mohseni S, Zarei N, Ragan ED (2021). A multidisciplinary survey and framework for design and evaluation of explainable AI systems. ACM Trans. Interactive Intell. Syst..

[39] Chen, J., Kallus, N., Mao, X., Svacha, G. & Udell, M. Fairness under unawareness: Assessing disparity when protected class is unobserved. In *Proceedings of the conference on fairness, accountability, and transparency*, 339–348 (2019).

[40] Datta, A., Tschantz, M. C. & Datta, A. Automated experiments on ad privacy settings: A tale of opacity, choice, and discrimination. preprint at https://arxiv.org/abs/1408.6491 (2014).

[41] Leslie, D. Understanding artificial intelligence ethics and safety: A guide for the responsible design and implementation of ai systems in the public sector. Available at *SSRN 3403301* (2019).

[42] Sokol, K. & Flach, P. Explainability fact sheets: a framework for systematic assessment of explainable approaches. In *Proceedings of the 2020 Conference on Fairness, Accountability, and Transparency*, 56–67 (2020).

[43] Liu X, Rivera SC, Moher D, Calvert MJ, Denniston AK (2020). Reporting guidelines for clinical trial reports forinterventions involving artificial intelligence: the consort-AI extension. BMJ.

[44] DECIDE-AI Steering Group. (2021). DECIDE-AI: new reporting guidelines to bridge the development-to-implementation gap in clinical artificial intelligence. Nat. Med..

[45] Cabitza, F. & Campagner, A. The need to separate the wheat from the chaff in medical informatics: Introducing a comprehensive checklist for the (self)-assessment of medical ai studies (2021). 10.1016/j.ijmedinf.2021.104510 34108105

[46] Hernandez-Boussard T, Bozkurt S, Ioannidis JP, Shah NH (2020). Minimar (minimum information for medical ai reporting): developing reporting standards for artificial intelligence in health care. J. Am. Med. Inf. Assoc..

[47] Scott, I., Carter, S. & Coiera, E. Clinician checklist for assessing suitability of machine learning applications in healthcare. *BMJ Health & Care Informatics* **28** (2021). 10.1136/bmjhci-2020-100251 PMC7871244 33547086

[48] Tjoa E, Guan C (2020). A survey on explainable artificial intelligence (xai): Toward medical xai. IEEE Trans. Neural Netw. Learn. Syst..

[49] van der Velden BH, Kuijf HJ, Gilhuijs KG, Viergever MA (2022). Explainable artificial intelligence (xai) in deep learning-based medical image analysis. Med. Image Anal.

[50] Gulum MA, Trombley CM, Kantardzic M (2021). A review of explainable deep learning cancer detection models in medical imaging. Appl. Sci..

[51] Krizhevsky A, Sutskever I, Hinton GE (2017). Imagenet classification with deep convolutional neural networks. Commun. ACM.

[52] Moher D, Liberati A, Tetzlaff J, Altman DG, Group P (2009). Preferred reporting items for systematic reviews and meta-analyses: the prisma statement. PLoS Med..

[53] Rudin C (2019). Stop explaining black box machine learning models for high stakes decisions and use interpretable models instead. Nat. Mach. Intell..

[54] Molnar, C. *Interpretable machine learning* (Lulu. com, 2020).

[55] Abdel Magid S (2020). Channel Embedding for Informative Protein Identification from Highly Multiplexed Images. 23rd International Conference on Medical Image Computing and Computer-Assisted Intervention, MICCAI 2020, October 4, 2020 - October 8, 2020.

[56] Afshar, P. et al. MIXCAPS: A capsule network-based mixture of experts for lung nodule malignancy prediction. *Pattern Recognition* **116**, 10.1016/j.patcog.2021.107942NS (2021).

[57] Fan M, Chakraborti T, Chang EIC, Xu Y, Rittscher J (2020). Microscopic Fine-Grained Instance Classification Through Deep Attention. 23rd International Conference on Medical Image Computing and Computer-Assisted Intervention, MICCAI 2020, October 4, 2020 - October 8, 2020.

[58] Graziani M, Lompech T, Muller H, Depeursinge A, Andrearczyk V (2020). Interpretable CNN Pruning for Preserving Scale-Covariant Features in Medical Imaging. 3rd International Workshop on Interpretability of Machine Intelligence in Medical Image Computing, iMIMIC 2020, the 2nd International Workshop on Medical Image Learning with Less Labels and Imperfect Data, MIL3ID 2020, and the 5th International Workshop o.

[59] An, F., Li, X. & Ma, X. Medical Image Classification Algorithm Based on Visual Attention Mechanism-MCNN. *Oxidative Medicine and Cellular Longevity* **2021**, https://www.embase.com/search/results?subaction=viewrecord&id=L2011217895&from=export10.1155/2021/6280690 (2021). 10.1155/2021/6280690 PMC7914083 33688390

[60] He, S. et al. Multi-channel attention-fusion neural network for brain age estimation: Accuracy, generality, and interpretation with 16,705 healthy MRIs across lifespan. *Med. Image Anal.* **72**, https://www.embase.com/search/results?subaction=viewrecord&id=L2012117928&from=export10.1016/j.media.2021.102091 (2021). 10.1016/j.media.2021.102091 PMC8316301 34038818

[61] Hou, B., Kang, G., Xu, X. & Hu, C. Cross Attention Densely Connected Networks for Multiple Sclerosis Lesion Segmentation. *2019 IEEE International Conference on Bioinformatics and Biomedicine, BIBM 2019, November 18, 2019 - November 21, 2019* 2356–2361, 10.1109/BIBM47256.2019.8983149NS (2019).

[62] Huang Y, Chung ACS (2019). Evidence localization for pathology images using weakly supervised learning. 22nd International Conference on Medical Image Computing and Computer-Assisted Intervention, MICCAI 2019, October 13, 2019 - October 17, 2019.

[63] Morvan L (2020). Learned Deep Radiomics for Survival Analysis with Attention. 3rd International Workshop on Predictive Intelligence in Medicine, PRIME 2020, held in conjunction with the Medical Image Computing and Computer Assisted Intervention, MICCAI 2020, October 8, 2020 - October 8, 2020.

[64] Saleem, H., Shahid, A. R. & Raza, B. Visual interpretability in 3D brain tumor segmentation network. *Comput. Biology Med.* **133**, https://www.embase.com/search/results?subaction=viewrecord&id=L2011734982&from=export10.1016/j.compbiomed.2021.104410 (2021). 10.1016/j.compbiomed.2021.104410 33894501

[65] Shahamat H, Saniee Abadeh M (2020). Brain MRI analysis using a deep learning based evolutionary approach. Neural Netw..

[66] Singla S (2018). Subject2Vec: generative-discriminative approach from a set of image patches to a vector. Med. Image Comput. Comput. Assist Interv..

[67] Sun J, Darbehani F, Zaidi M, Wang B (2020). SAUNet: Shape Attentive U-Net for Interpretable Medical Image Segmentation. 23rd International Conference on Medical Image Computing and Computer-Assisted Intervention, MICCAI 2020, October 4, 2020 - October 8, 2020.

[68] Xu X (2021). Automatic glaucoma detection based on transfer induced attention network. Biomed. Eng. Online.

[69] Yang H, Kim J-Y, Kim H, Adhikari SP (2020). Guided soft attention network for classification of breast cancer histopathology images. IEEE Trans. Med. Imaging.

[70] Diao, J. A. et al. Human-interpretable image features derived from densely mapped cancer pathology slides predict diverse molecular phenotypes. *Nat. Commun.* **12**, https://www.embase.com/search/results?subaction=viewrecord&id=L2010776995&from=export10.1038/s41467-021-21896-9 (2021). 10.1038/s41467-021-21896-9 PMC7955068 33712588

[71] Dong, Y. et al. A Polarization-imaging-based machine learning framework for quantitative pathological diagnosis of cervical precancerous lesions. *IEEE Trans. Med. Imaging*. https://www.embase.com/search/results?subaction=viewrecord&id=L635538309&from=export10.1109/TMI.2021.3097200 (2021). 10.1109/TMI.2021.3097200 34260351

[72] Giannini V, Rosati S, Regge D, Balestra G (2016). Texture features and artificial neural networks: A way to improve the specificity of a CAD system for multiparametric MR prostate cancer. 14th Mediterranean Conference on Medical and Biological Engineering and Computing, MEDICON 2016, March 31, 2016 - April 2, 2016.

[73] Loveymi S, Dezfoulian MH, Mansoorizadeh M (2020). Generate structured radiology report from CT images using image annotation techniques: preliminary results with liver CT. J. Dig. Imaging.

[74] MacCormick, I. J. C. et al. Accurate, fast, data efficient and interpretable glaucoma diagnosis with automated spatial analysis of the whole cup to disc profile. *PLoS ONE* **14**, https://www.embase.com/search/results?subaction=viewrecord&id=L625837308&from=export10.1371/journal.pone.0209409 (2019). 10.1371/journal.pone.0209409 PMC6328156 30629635

[75] Kunapuli G (2018). A decision-support tool for renal mass classification. J. Digit. Imaging.

[76] Shen T, Wang J, Gou C, Wang F-Y (2020). Hierarchical fused model with deep learning and type-2 fuzzy learning for breast cancer diagnosis. IEEE Trans. Fuzzy Syst..

[77] Li, J., Shi, H. & Hwang, K.-S. An explainable ensemble feedforward method with Gaussian convolutional filter. *Knowl.-Based Syst.* **225**, 10.1016/j.knosys.2021.107103NS (2021).

[78] Puyol-Anton E (2020). Assessing the impact of blood pressure on cardiac function using interpretable biomarkers and variational autoencoders. 10th International Workshop on Statistical Atlases and Computational Models of the Heart, STACOM 2019, held in conjunction with the 22nd International Conference on Medical Image Computing and Computer Assisted Intervention, MICCAI 2019, October 13, 2019.

[79] Wongvibulsin S, Wu KC, Zeger SL (2020). Improving clinical translation of machine learning approaches through clinician-tailored visual displays of black box algorithms: development and validation. JMIR Med. Inform..

[80] Lin, Y., Wei, L., Han, S. X., Aberle, D. R. & Hsu, W. EDICNet: An end-to-end detection and interpretable malignancy classification network for pulmonary nodules in computed tomography. *Medical Imaging 2020: Computer-Aided Diagnosis, February 16, 2020 - February 19, 2020* **11314**, The Society of Photo–Optical Instrumentation Engin. 10.1117/12.2551220NS (2020). 10.1117/12.2551220 PMC7325481 32606487

[81] Kim, S. T., Lee, H., Kim, H. G. & Ro, Y. M. ICADx: Interpretable computer aided diagnosis of breast masses. *Medical Imaging 2018: Computer-Aided Diagnosis, February 12, 2018 - February 15, 2018* **10575**, DECTRIS Ltd.; The Society of Photo–Optical Instrum. 10.1117/12.2293570NS (2018).

[82] Kim ST, Lee J-H, Lee H, Ro YM (2018). Visually interpretable deep network for diagnosis of breast masses on mammograms. Phys. Med. Biology.

[83] Puyol-Antón E (2020). Interpretable deep models for cardiac resynchronisation therapy response prediction. Med. Image Comput. Comput. Assist Interv.

[84] Wang CJ (2019). Deep learning for liver tumor diagnosis part II: convolutional neural network interpretation using radiologic imaging features. Eur. Radiol..

[85] Codella NCF (2018). Collaborative human-AI (CHAI): Evidence-based interpretable melanoma classification in dermoscopic images. 1st International Workshop on Machine Learning in Clinical Neuroimaging, MLCN 2018, 1st International Workshop on Deep Learning Fails, DLF 2018, and 1st International Workshop on Interpretability of Machine Intelligence in Medical Image Computing, iMIMIC.

[86] Barata, C., Celebi, M. E. & Marques, J. S. Explainable skin lesion diagnosis using taxonomies. *Pattern Recognition* **110**, 10.1016/j.patcog.2020.107413NS (2021).

[87] Silva W, Fernandes K, Cardoso MJ, Cardoso JS (2018). Towards complementary explanations using deep neural networks. 1st International Workshop on Machine Learning in Clinical Neuroimaging, MLCN 2018, 1st International Workshop on Deep Learning Fails, DLF 2018, and 1st International Workshop on Interpretability of Machine Intelligence in Medical Image Computing, iMIMIC.

[88] Khaleel M, Tavanapong W, Wong J, Oh J, De Groen P (2021). Hierarchical visual concept interpretation for medical image classification. 34th IEEE International Symposium on Computer-Based Medical Systems, CBMS 2021, June 7, 2021 - June 9, 2021.

[89] Pereira S (2018). Enhancing interpretability of automatically extracted machine learning features: application to a RBM-Random Forest system on brain lesion segmentation. Med. Image Anal..

[90] Yan, K. et al. Holistic and comprehensive annotation of clinically significant findings on diverse CT images: Learning from radiology reports and label ontology. *32nd IEEE/CVF Conference on Computer Vision and Pattern Recognition, CVPR 2019, June 16, 2019 - June 20, 2019* 2019-June, 8515–8524, 10.1109/CVPR.2019.00872NS (2019).

[91] Chen H, Miao S, Xu D, Hager GD, Harrison AP (2020). Deep hiearchical multi-label classification applied to chest x-ray abnormality taxonomies. Med. Image Anal..

[92] Verma A, Shukla P, Verma S (2019). An interpretable SVM based model for cancer prediction in mammograms. 1st International Conference on Communication, Networks and Computing, CNC 2018, March 22, 2018 - March 24, 2018.

[93] Li Y (2020). Computer-aided cervical cancer diagnosis using time-lapsed colposcopic images. IEEE Trans. Med. Imaging.

[94] Wang K (2019). A dual-mode deep transfer learning (D2TL) system for breast cancer detection using contrast enhanced digital mammograms. IISE Trans. Healthcare Syst. Eng..

[95] Zhao G, Zhou B, Wang K, Jiang R, Xu M (2018). Respond-CAM: Analyzing deep models for 3D imaging data by visualizations. 21st International Conference on Medical Image Computing and Computer Assisted Intervention, MICCAI 2018, September 16, 2018 - September 20, 2018.

[96] Folke, T., Yang, S. C.-H., Anderson, S. & Shafto, P. Explainable AI for medical imaging: Explaining pneumothorax diagnoses with Bayesian teaching. *Artificial Intelligence and Machine Learning for Multi-Domain Operations Applications III 2021, April 12, 2021 - April 16, 2021* **11746**, The Society of Photo–Optical Instrumentation Engin. 10.1117/12.2585967NS (2021).

[97] Liao W (2020). Clinical interpretable deep learning model for glaucoma diagnosis. IEEE J. Biomed. Health Inf..

[98] Shinde S, Chougule T, Saini J, Ingalhalikar M (2019). HR-CAM: Precise localization of pathology using multi-level learning in CNNS. 22nd International Conference on Medical Image Computing and Computer-Assisted Intervention, MICCAI 2019, October 13, 2019 - October 17, 2019.

[99] Ballard DH (1987). Modular learning in neural networks. AAAI.

[100] Biffi C (2018). Learning interpretable anatomical features through deep generative models: Application to cardiac remodeling. 21st International Conference on Medical Image Computing and Computer Assisted Intervention, MICCAI 2018, September 16, 2018 - September 20, 2018.

[101] Couteaux V, Nempont O, Pizaine G, Bloch I (2019). Towards interpretability of segmentation networks by analyzing deepDreams. 2nd International Workshop on Interpretability of Machine Intelligence in Medical Image Computing, iMIMIC 2019, and the 9th International Workshop on Multimodal Learning for Clinical Decision Support, ML-CDS 2019, held in conjunction with the 22nd Interna.

[102] Guo X (2016). Intelligent medical image grouping through interactive learning. Int. J. Data Sci. Anal..

[103] Janik, A., Dodd, J., Ifrim, G., Sankaran, K. & Curran, K. Interpretability of a deep learning model in the application of cardiac MRI segmentation with an ACDC challenge dataset. *Medical Imaging 2021: Image Processing, February 15, 2021 - February 19, 2021* 11596, The Society of Photo–Optical Instrumentation Engin. 10.1117/12.2582227NS (2021).

[104] Sari CT, Gunduz-Demir C (2019). Unsupervised feature extraction via deep learning for histopathological classification of colon tissue images. IEEE Trans. Med. Imaging.

[105] Venugopalan J, Tong L, Hassanzadeh HR, Wang MD (2021). Multimodal deep learning models for early detection of Alzheimer’s disease stage. Sci. Rep..

[106] Zhu P, Ogino M (2019). Guideline-based additive explanation for computer-aided diagnosis of lung nodules. 2nd International Workshop on Interpretability of Machine Intelligence in Medical Image Computing, iMIMIC 2019, and the 9th International Workshop on Multimodal Learning for Clinical Decision Support, ML-CDS 2019, held in conjunction with the 22nd Interna.

[107] Pirovano A, Heuberger H, Berlemont S, Ladjal S, Bloch I (2020). Improving interpretability for computer-aided diagnosis tools on whole slide imaging with multiple instance learning and gradient-based explanations. 3rd International Workshop on Interpretability of Machine Intelligence in Medical Image Computing, iMIMIC 2020, the 2nd International Workshop on Medical Image Learning with Less Labels and Imperfect Data, MIL3ID 2020, and the 5th International Workshop o.

[108] Hao J, Kosaraju SC, Tsaku NZ, Song DH, Kang M (2020). PAGE-Net: interpretable and integrative deep learning for survival analysis using histopathological images and genomic data. Pacific Symposium on Biocomputing. Pacific Symposium on Biocomputing.

[109] de Sousa IP, Vellasco MMBR, da Silva EC (2020). Approximate explanations for classification of histopathology patches. Workshops of the 20th Joint European Conference on Machine Learning and Knowledge Discovery in Databases, ECML PKDD, September 14, 2020 - September 18, 2020.

[110] Li X, Dvornek NC, Zhuang J, Ventola P, Duncan JS (2018). Brain biomarker interpretation in ASD using deep learning and fMRI. 21st International Conference on Medical Image Computing and Computer Assisted Intervention, MICCAI 2018, September 16, 2018 - September 20, 2018.

[111] Quellec, G. et al. ExplAIn: Explanatory artificial intelligence for diabetic retinopathy diagnosis. *Med. Image Anal.* **72**, https://www.embase.com/search/results?subaction=viewrecord&id=L2012995582&from=export10.1016/j.media.2021.102118 (2021). 10.1016/j.media.2021.102118 34126549

[112] Uzunova, H., Ehrhardt, J., Kepp, T. & Handels, H. Interpretable explanations of black box classifiers applied on medical images by meaningful perturbations using variational autoencoders. *Medical Imaging 2019: Image Processing, February 19, 2019 - February 21, 2019* **10949**, The Society of Photo–Optical Instrumentation Engin. 10.1117/12.2511964NS (2019).

[113] Liu J (2019). Ultrasound liver fibrosis diagnosis using multi-indicator guided deep neural networks. 10th International Workshop on Machine Learning in Medical Imaging, MLMI 2019 held in conjunction with the 22nd International Conference on Medical Image Computing and Computer-Assisted Intervention, MICCAI 2019, October 13, 2019 - October 13, 2019.

[114] Liu, Y. et al. Act like a radiologist: towards reliable multi-view correspondence reasoning for mammogram mass detection. *IEEE Trans. Pattern Anal. Mach. Intell.*10.1109/TPAMI.2021.3085783NS (2021). 10.1109/TPAMI.2021.3085783 34061740

[115] Oktay O (2018). Anatomically constrained neural networks (ACNNs): application to cardiac image enhancement and segmentation. IEEE Trans. Med. Imaging.

[116] Peng T, Boxberg M, Weichert W, Navab N, Marr C (2019). Multi-task learning of a deep K-nearest neighbour network for histopathological image classification and retrieval. 22nd International Conference on Medical Image Computing and Computer-Assisted Intervention, MICCAI 2019, October 13, 2019 - October 17, 2019.

[117] Liu, Y., Li, Z., Ge, Q., Lin, N. & Xiong, M. Deep Feature Selection and Causal Analysis of Alzheimer’s Disease. *Front. Neurosci.* **13**, https://www.embase.com/search/results?subaction=viewrecord&id=L629992085&from=export10.3389/fnins.2019.01198 (2019). 10.3389/fnins.2019.01198 PMC6872503 31802999

[118] Ren H (2021). Interpretable pneumonia detection by combining deep learning and explainable models with multisource data. IEEE Access.

[119] Velikova M, Lucas PJF, Samulski M, Karssemeijer N (2013). On the interplay of machine learning and background knowledge in image interpretation by Bayesian networks. Artif. Intell. Med..

[120] Carneiro, G., Zorron Cheng Tao Pu, L., Singh, R. & Burt, A. Deep learning uncertainty and confidence calibration for the five-class polyp classification from colonoscopy. *Med. Image Anal.* **62**, https://www.embase.com/search/results?subaction=viewrecord&id=L2005189093&from=export10.1016/j.media.2020.101653 (2020). 10.1016/j.media.2020.101653 32172037

[121] Sabol, P. et al. Explainable classifier for improving the accountability in decision-making for colorectal cancer diagnosis from histopathological images. *J. Biomed. Inf.* **109**, https://www.embase.com/search/results?subaction=viewrecord&id=L2007460563&from=export10.1016/j.jbi.2020.103523 (2020). 10.1016/j.jbi.2020.103523 32758538

[122] Tanno, R. et al. Uncertainty modelling in deep learning for safer neuroimage enhancement: Demonstration in diffusion MRI. *NeuroImage* **225**, https://www.embase.com/search/results?subaction=viewrecord&id=L2008373754&from=export10.1016/j.neuroimage.2020.117366 (2021). 10.1016/j.neuroimage.2020.117366 33039617

[123] Doshi-Velez, F. & Kim, B. Towards a rigorous science of interpretable machine learning. preprint athttps://arxiv.org/abs/1702.08608 (2017).

[124] Adebayo, J. et al. Sanity checks for saliency maps. In Bengio, S. et al. (eds.) *Advances in Neural Information Processing Systems*, vol. 31, https://proceedings.neurips.cc/paper/2018/file/294a8ed24b1ad22ec2e7efea049b8737-Paper.pdf (Curran Associates, Inc., 2018).

[125] Yeche H, Harrison J, Berthier T (2019). UBS: A dimension-agnostic metric for concept vector interpretability applied to radiomics. 2nd International Workshop on Interpretability of Machine Intelligence in Medical Image Computing, iMIMIC 2019, and the 9th International Workshop on Multimodal Learning for Clinical Decision Support, ML-CDS 2019, held in conjunction with the 22nd Interna.

[126] Chen, H., Miao, S., Xu, D., Hager, G. D. & Harrison, A. P. Deep hierarchical multi-label classification of chest x-ray images. In *International Conference on Medical Imaging with Deep Learning*, 109–120 (PMLR, 2019).

[127] Zhang, Z. et al. Origa-light: An online retinal fundus image database for glaucoma analysis and research. In *2010 Annual International Conference of the IEEE Engineering in Medicine and Biology*, 3065–3068 (IEEE, 2010). 10.1109/IEMBS.2010.5626137 21095735

[128] Menze BH (2014). The multimodal brain tumor image segmentation benchmark (brats). IEEE Trans. Med. Imaging.

