# Supplementary material for: Explainable medical imaging AI needs human-centered design: guidelines and evidence from a systematic review
Source: NPJ Digit Med. 2022 Oct 19;5:156. doi: 10.1038/s41746-022-00699-2 (PMC9581990; doi:10.1038/s41746-022-00699-2)
Supplement: Supplementary file 1 — Supplementary Information of the main article [file 41746_2022_699_MOESM1_ESM.pdf]

A The INTRPRT guideline: A case study

To contextualize the *INTRPRT guideline*, we present case studies to demonstrate the envisioned use. Because none of the surveyed papers take into account all aspects mentioned in the *INTRPRT guideline*, we include three published papers that are representative of different parts of the *INTRPRT guideline*. Case 1 (C1): Xie et al. [15] focused on the formative user research stage to determine physicians' needs when exploring and understanding an Artificial Intelligence (AI)-generated analysis report in the context of chest X-rays diagnosis. Through surveys, a co-design process, and user evaluations, different features of the explanations that physicians expect when they interact with the system were identified. Case 2 (C2): Motivated by the fact that the radiologists characterize breast masses according to Breast Imaging Reporting and Data System (BI-RADS) (public evidence), Kim et al. [82] directly encoded the BI-RADS characteristics of breast masses to build a deep learning model for mass classification. Case 3 (C3): Sabol et al. [121] created an explainable system for colorectal cancer diagnosis from histopathological images by providing human-friendly explanations for a certain prediction. The validation of the system included the assessment of transparency through a human factors evaluation with 14 pathologists who interacted with the system through a graphical user interface.

We identify which aspects within the three cases follow the *INTRPRT guideline*:

G1: Specify the clinical scenario, constraints, requirements, and end users.
In C1, Machine Learning (ML) designers determined the requirements in current clinical practice by conducting paired surveys on physicians and radiologists, where the latter provide a diagnosis and the former have to interpret the results. The target users (referring physicians) were identified within the context in which the system will be used.

G2: Justify the choice of transparency and determine the level of evidence.
In C1, to inform the design of AI-enabled chest X-ray analysis, iteratively developed evidence was generated by involving potential end users of the system through interactions with low and high-fidelity prototypes. The design choices to build the prototypes were based on the initial needs identified with a survey of explanations between referring physicians and radiologists.
In C2, the acBI-RADS standard serves as public evidence because it is widely accepted and applied among clinicians for mass classification.

G3: Clarify how the model follows the justification of transparency.
In C2, the public evidence, namely BI-RADS characteristics of breast masses, are encoded as features and concatenated with deep encoded image features for the final tissue classification. As a result, BI-RADS characteristics are explicitly extracted and have direct impact in the final decision making.

G4: Determine how to communicate with end users.
In C1, a graphical interface was implemented as a high-fidelity prototype. The interface presented one patient's case at a time, displaying the Chest X-ray (CXR) image, the significant observations generated from an AI model (as textual labels), and eight explanations features that were manually generated, such as highlighting evidence towards a specific diagnosis in the image, probabilities for each possible conclusion or comparisons with previous patients' cases.
C3 created a graphical user interface that showed the original image of the Whole Slide Image (WSI) and the corresponding label map with a color code for different tissue types. Pathologists can examine an arbitrary area of the WSI by clicking on the desired area. Subsequently, the outcomes of the system were displayed, including the prediction result with a semantical explanation. Besides, a visualization of the training image most similar to the current one as well as training images with other tissue types are presented as references and support for the prediction.

G5: Report task performance of the ML systems.
In C2, quantitative results of the method with/without implementing BI-RADS characteristics are presented on a public mammogram database. Both accuracy and Area under the ROC Curve (AUC) are used as evaluation metrics of the model itself.
C3 reported the accuracy of the classifier in a class-balanced dataset of tissue slides.

G6: Assess correctness and human factors of system transparency.
C3 involved 14 pathologists to evaluate four human factors of the system: usefulness, level of detail, reliability, and experience quality. Pathologists were first asked to examine 20 arbitrary areas in a

graphical user interface and evaluate the prediction outcome. At the end of the experiment session, every participant was asked to fill out a questionnaire based on their perception of the system.

Lastly, we present examples on where and how authors in the three cases could integrate the use of the *INTRPRT guideline* in their articles:

C1: At the beginning of the Method section: "We were primarily concerned on the user-centered iterative design of a proof-of-concept system prototype of an AI-based medical image analysis tool with different explanation features to assist physicians. The outcome of the user-centered empirical formative research approach includes recommendations and insights that may benefit future development of explainable algorithms for medical image analysis."

C2: In the Method section: "We followed the widely accepted BI-RADS standard for breast mass assessment as a readily interpretable backbone as a basis for our deep network." In the Results section: "The verification of the system included a task performance comparison against a model without the interpretable BI-RADS component on a public database. Additional qualitative visualizations showed relevant areas where the model exploited more information."

C3: At the end of the Introduction section: "We developed and discussed the mathematical structure of a classifier that provides different outputs to explain the plausibility of the decision. A quantitative evaluation of the system's performance was conducted on a public database. We also report the system's acceptability assessed through a user study with 14 pathologists."

B Search strategy

We use the following search term for PubMed and EMBASE to screen titles, abstracts, and keywords of all available records:

("interpretable" OR "explainable" OR "interpretability" OR "explainability" OR "interpretation" OR "explanation" OR "interpreting" OR "explaining" OR "interpret" OR "explain") AND ("artificial intelligence" OR "deep learning" OR "machine learning" OR "neural network") AND ("image" OR "imaging") AND ("healthcare" OR "health care" OR "clinical" OR "medical")

In addition to the above search terms, Compendex offers "controlled terms" to better locate desired articles. We first filter all records with the following search term for titles, abstracts, and keywords:

(interpret* OR explain* OR "explanation")

Then we use "controlled terms" to further filter all the remaining records:

("artificial intelligence" OR "neural networks" OR "machine learning" OR "deep learning" OR "deep neural networks" OR "convolutional neural networks" OR "learning systems" OR "supervised learning" OR "network architecture") AND ("medical imaging" OR "medical image processing" OR "medical computing" OR "tissue")

For screening in all three databases, we also exclude articles with ("survey" OR "review") in the tile and ("workshop") in the title and abstract.

C Details of screening and full-text review

The initial search resulted in 2508 records, and after removal of duplicates, 1731 unique studies were included for screening. During screening, 1514 articles were excluded because they 1) were not transparent methods (n=947); 2) were not imaging ML methods (n=422); 3) only had simple visualization explanations (n=129) and 4) were not for medical problems (n=19). We found unsubstantiated claims around transparency with only simple visualizations such as Class Activation Maps (CAMs) widely occur in ML methods or using existing transparent methods. As a result, we excluded them and focused on articles with other transparent ML methods. The remaining 217 articles were included in full-text review. In total, 149 records were further excluded because they 1) used exactly the same transparency mechanism as previously proposed work for natural image problems (n=48); 2) were not transparent methods (n=43); 3) were not long articles and therefore could not be analyzed with the required detail (n=24); 4) were not imaging ML methods (n=21); 5) were not for medical problems (n=5); 6) did not have available full text (n=5); 7) were repeated works (n=3). The criterion for long

articles we applied was single-column articles longer or equal to 8 pages or double-column articles longer or equal to 6 pages, excluding reference pages.

D Data extraction

Supplementary Table 1 describes in detail our data extraction approach for the studies included in this review. Supplementary Tables 2 and 3 present the details of each study included in the review in the design preparation and implementation, respectively.

Supplementary Table 1: Data extraction strategy related to the six themes.

Theme	Item	Description
Incorporation	Clinician engineering team	Any clinical stakeholders part of the study team and author list
	Formative user research	Any technique to understand the target population
Target	End users	Users of the systems
Prior	Justification of transparency	Description of the choice of transparency
	Prior type	Computer vision / clinical knowledge prior
Task	Inputs & outputs	Task inputs & outputs
	Task difficulty	Routine / super-human tasks
Interpretability	Technical mechanism	Explicit transparency technique
	Transparency type	Interpretable (provides its own explanations) / Explainable (needs post-hoc explanations)
Reporting	Metrics	Task performance evaluation and transparency assessment metrics
	Transparency performance	Performance against comparable baseline models
	Incorporation performance	Performance of human-AI incorporation against AI alone
	Human subjects	Number of end users involved in evaluation

Supplementary Table 2: A summary of transparency design preparation details of each study reviewed. The definition of each column is the same as in Supplementary Table 1.

Study ID	Author team	End users	Task type	Formative user research	Task difficulty	Clear justification of transparency	Prior type
[55]	Yes	Not specified	Prediction	None	Routine	Yes	Computer vision
[56]	Yes	Decision providers	Prediction	None	Routine	No	Computer vision
[85]	Yes	Decision providers	Prediction	None	Routine	No	Clinical knowledge
[101]	No	Not specified	Segmentation	None	Routine	Yes	Computer vision
[109]	No	Not specified	Prediction	None	Routine	Yes	Computer vision
[70]	Yes	Decision providers	Prediction	None	Super-human	Yes	Clinical knowledge
[71]	Yes	Decision providers	Prediction	None	Routine	Yes	Clinical knowledge

[57]	Yes	Not specified	Prediction	None	Routine	No	Computer vision
[96]	No	Decision providers	Prediction	None	Routine	Yes	Computer vision
[72]	Yes	Decision providers	Segmentation	None	Routine	No	Clinical knowledge
[58]	Cannot tell	Not specified	Prediction	None	Cannot tell	Yes	Computer vision
[58]	Yes	Not specified	Segmentation	None	Routine	Yes	Computer vision
[102]	Yes	Decision providers	Image grouping	None	Routine	Yes	Clinical knowledge
[59]	No	Not specified	Prediction	None	Routine	Yes	Computer vision
[86]	No	Decision providers	Prediction	None	Routine	Yes	Clinical knowledge
[100]	Yes	Not specified	Prediction	None	Routine	No	Computer vision
[120]	Yes	Decision providers	Prediction	None	Super-human	Yes	Computer vision
[108]	Yes	Decision providers	Prediction	None	Routine	Yes	Computer vision
[60]	Yes	Not specified	prediction	None	Cannot tell	Yes	Computer vision
[61]	Yes	Not specified	Segmentation	None	Routine	Yes	Computer vision
[62]	No	Decision providers	Prediction	None	Routine	Yes	Computer vision
[110]	Yes	Not specified	Prediction	None	Routine	Yes	Computer vision
[93]	Yes	Decision providers	Prediction	None	Routine	Yes	Clinical knowledge
[97]	No	Not specified	Prediction	None	Routine	Yes	Computer vision
[80]	Yes	Decision providers	Prediction	None	Routine	Yes	Clinical knowledge
[113]	Cannot tell	Not specified	Prediction	None	Routine	Yes	Clinical knowledge
[117]	No	Not specified	Prediction	None	Cannot tell	Yes	Clinical knowledge
[114]	No	Decision providers	Prediction	None	Routine	Yes	Clinical knowledge
[73]	No	Not specified	Predictions	None	Routine	No	Clinical knowledge
[74]	Yes	Not specified	Prediction	None	Routine	Yes	Clinical knowledge
[63]	Yes	Not specified	Prediction	None	Routine	Yes	Computer vision
[98]	Yes	Not specified	Prediction	None	Routine	Yes	Computer vision
[87]	Yes	Not specified	Prediction	None	Routine	Yes	Computer vision
[103]	Yes	Decision providers	Segmentation	None	Routine	Yes	Computer vision
[88]	Yes	Not specified	Prediction	None	Super-human	Yes	Computer vision
[81]	No	Decision providers	Prediction	None	Routine	Yes	Clinical knowledge
[82]	No	Decision providers	Prediction	None	Routine	Yes	Clinical knowledge
[75]	Yes	Not specified	prediction	None	Routine	Yes	Clinical knowledge

[121]	Yes	Decision providers	Prediction	None	Routine	Yes	Computer vision
[64]	No	Not specified	Segmentation	None	Routine	No	Computer vision
[104]	No	Not specified	prediction	None	Routine	No	Computer vision
[65]	No	Not specified	Prediction	None	Routine	Yes	Clinical knowledge
[76]	No	Not specified	Prediction	None	Routine	Yes	Clinical knowledge
[77]	No	Not specified	prediction	None	Routine	Yes	Computer vision
[115]	Yes	Not specified	Segmentation	None	Routine	Yes	Clinical knowledge
[116]	Yes	Decision providers	Prediction	None	Routine	Yes	Computer vision
[89]	Yes	Not specified	Segmentation	None	Routine	Yes	Computer vision
[112]	No	Decision providers	prediction	None	Routine	Yes	Computer vision
[107]	No	Decision providers	Prediction	None	Routine	No	Computer vision
[83]	No	Decision providers	Prediction	None	Super-human	Yes	Clinical knowledge
[78]	Yes	Decision providers	Prediction	None	Routine	Yes	Clinical knowledge
[111]	Yes	Decision providers	Prediction	None	Routine	No	Computer vision
[118]	Yes	Decision providers	prediction	None	Routine	No	Clinical knowledge
[66]	Yes	Not specified	Prediction	None	Routine	Yes	Computer vision
[67]	Yes	Not specified	Segmentation	None	Routine	Yes	Computer vision
[122]	Yes	Decision providers	Super-resolution	None	Super-human	Yes	Computer vision
[119]	Yes	Not specified	Prediction	None	Routine	Yes	Clinical knowledge
[105]	No	Decision providers	Prediction	None	Routine	Yes	Computer vision
[92]	No	Decision providers	prediction	None	Routine	Yes	Computer vision
[84]	Yes	Decision providers	Prediction	None	Routine	No	Clinical knowledge
[94]	Yes	Decision providers	Prediction	None	Routine	No	Computer vision
[79]	Yes	Decision providers	Prediction	None	Cannot tell	No	Clinical knowledge
[68]	Yes	Not specified	Prediction	None	Routine	Yes	Computer vision
[90]	Yes	Decision providers	Prediction	None	Routine	Yes	Clinical knowledge
[69]	Yes	Decision providers	Prediction	None	Routine	No	Computer vision
[125]	No	Decision providers	Prediction	None	Routine	Yes	Clinical knowledge
[95]	No	Not specified	Prediction	None	Routine	Yes	Computer vision
[106]	No	Not specified	Prediction	None	Routine	Yes	Clinical knowledge

Supplementary Table 3: A summary of transparency design implementation and validation details of each study reviewed. The definition of each column is the same as in Supplementary Table 1.

Study ID	Transparency type	Technical mechanism	Transparency assessment	Transparency performance	Incorporation performance	Human subjects
[55]	Interpretable	Attention	Spearman coefficients	Better	None	None
[56]	Interpretable	Attention	None	Better	None	None
[85]	Interpretable	Clustering	None	Better	None	None
[101]	Explainable	activation maximization	None	None	None	None
[109]	Explainable	Input perturbation	Visualization	None	None	None
[70]	Interpretable	Automatic extraction of clinically important features	Similarity	None	None	None
[71]	Interpretable	Automatic extraction of clinically important features	Visualization	None	None	None
[57]	Interpretable	Attention	None	Better	None	None
[96]	Explainable	Saliency maps	Level of trust	None	None	8
[72]	Interpretable	Hand-crafted feature computation	Confusion matrix	None	None	None
[58]	Interpretable	Network architecture pruning	Kappa, R^2 matrix	Better	None	None
[?]	Interpretable	Attention	Visualization	Better	None	None
[102]	Interpretable	Clustering	None	Better	Better	1
[59]	Interpretable	Attention	None	None	None	None
[86]	Interpretable	Decoded by simple transparent models	None	Comparable	None	None
[100]	Interpretable	Latent variable evolution	None	None	None	None
[120]	Interpretable	Uncertainty estimation / confidence calibration	None	Better	None	None
[108]	Interpretable	Ranking of features	C-index, visualization	Better	None	None
[60]	Interpretable	Attention	Pearson correlation, visualization	Better	None	None
[61]	Interpretable	Attention	None	Better	None	None
[62]	Interpretable	Attention	None	Better	None	None
[110]	Explainable	Input corruption	Visualization	None	None	None
[93]	Interpretable	Decoded by simple transparent models	T-SNE visualization	Better	None	None
[97]	Explainable	Visualization	Visualization	Better	None	None
[80]	Interpretable	Automatic extraction of clinically important features	None	None	None	None

[113]	Interpretable	Network structure modification	None	Better	None	None
[117]	Interpretable	Causal inference	Causal relationships, visualization	None	None	None
[114]	Interpretable	Network structure modification	Visualization	Better	None	None
[73]	Interpretable	Automatic extraction of clinically important features	None	Better	None	None
[74]	Interpretable	Automatic extraction of clinically important features	None	None	None	None
[63]	Interpretable	Attention	C-index	Better	None	None
[98]	Explainable	Class activation mapping	Visualization	Better	None	None
[87]	Explainable	Decoded by simple transparent models	Correctness, completeness and compactness	None	None	None
[103]	Interpretable	Clustering	None	None	None	None
[88]	Interpretable	Decoded by simple transparent models	Faithfulness, relevance scores	None	None	None
[81]	Interpretable	Hand-crafted feature computation	None	Better	None	None
[82]	Interpretable	Automatic extraction of clinically important features	Visualization	Better	None	None
[75]	Interpretable	Relation analysis	None	Better	None	None
[121]	Interpretable	Uncertainty estimation / confidence calibration	Certainty rate & error, User perception	Comparable	None	14
[64]	Explainable	Attention	Deletion metric	None	None	None
[104]	Interpretable	Clustering	Visualization	Better	None	None
[65]	Interpretable	Attention with domain knowledge	None	Better	None	None
[76]	Interpretable	Decoded by simple transparent models	None	Better	None	None
[77]	Interpretable	Representative feature extraction	None	Better	None	None
[115]	Interpretable	Prior knowledge into latent space	Agreement	Better	None	2
[116]	Interpretable	Image retrieval	Visualization	Better	None	None

[89]	Explainable	Decoded by simple transparent models	Visualization	Comparable	None	None
[112]	Interpretable	Perturbation analysis	Visualization	Better	None	None
[107]	Explainable	Feature importance analysis	Visualizations	None	None	None
[83]	Interpretable	Network structure modification	Visualization	Comparable	None	None
[78]	Interpretable	Relation analysis	R^2 matrix	None	None	None
[111]	Interpretable	Automatic extraction of clinically important features	Kappa	Better	None	None
[118]	Interpretable	Causal inference	None	Comparable	None	None
[66]	Interpretable	Attention	R^2 matrix, visualization	Better	None	None
[67]	Interpretable	Attention	Visualization	Better	None	None
[122]	Interpretable	Uncertainty estimation / confidence calibration	Reliability	Better	None	None
[119]	Interpretable	Causal inference	None	None	None	None
[105]	Explainable	Perturbation analysis	None	None	None	None
[92]	Interpretable	Decoded by simple transparent models	Visualization	None	None	None
[84]	Interpretable	Automatic extraction of clinically important features	None	None	None	None
[94]	Explainable	Visualization	Visualization	None	None	None
[79]	Explainable	Relation analysis	Visualization	None	None	None
[68]	Interpretable	Attention	Visualization	Better	None	None
[90]	Interpretable	Decoded by simple transparent models	Visualization	Better	None	None
[69]	Interpretable	Attention	Visualization	Better	None	None
[125]	Interpretable	Concept's importance analysis	R^2 matrix	None	None	None
[95]	Explainable	Backpropagation guidance	Visualization, Kendall's Tau metric	None	None	None
[106]	Explainable	Perturbation analysis	Shapley values	None	None	None

Supplementary References

[1] Topol, E. J. High-performance medicine: the convergence of human and artificial intelligence. *Nature Medicine* **25**, 44–56 (2019).

[2] Obermeyer, Z., Powers, B., Vogeli, C. & Mullainathan, S. Dissecting racial bias in an algorithm used to manage the health of populations. *Science* **366**, 447–453 (2019).

[3] Ghassemi, M., Oakden-Rayner, L. & Beam, A. L. The false hope of current approaches to explainable artificial intelligence in health care. *The Lancet Digital Health* **3**, e745–e750 (2021).

[4] McCoy, L. G., Brenna, C. T., Chen, S. S., Vold, K. & Das, S. Believing in black boxes: Machine learning for healthcare does not need explainability to be evidence-based. *Journal of Clinical Epidemiology* **142**, 252–257 (2022).

[5] Vellido, A. The importance of interpretability and visualization in machine learning for applications in medicine and health care. *Neural Computing and Applications* **32**, 18069–18083 (2020).

[6] Char, D. S., Abràmoff, M. D. & Feudtner, C. Identifying ethical considerations for machine learning healthcare applications. *The American Journal of Bioethics* **20**, 7–17 (2020).

[7] Holzinger, A., Langs, G., Denk, H., Zatloukal, K. & Müller, H. Causability and explainability of artificial intelligence in medicine. *Wiley Interdisciplinary Reviews: Data Mining and Knowledge Discovery* **9**, e1312 (2019).

[8] Markus, A. F., Kors, J. A. & Rijnbeek, P. R. The role of explainability in creating trustworthy artificial intelligence for health care: a comprehensive survey of the terminology, design choices, and evaluation strategies. *Journal of Biomedical Informatics* **113**, 103655 (2021).

[9] Salahuddin, Z., Woodruff, H. C., Chatterjee, A. & Lambin, P. Transparency of deep neural networks for medical image analysis: A review of interpretability methods. *Computers in biology and medicine* **140**, 105111 (2022).

[10] Banegas-Luna, A. J. *et al.* Towards the interpretability of machine learning predictions for medical applications targeting personalised therapies: A cancer case survey. *International Journal of Molecular Sciences* **22**, 4394 (2021).

[11] Ploug, T. & Holm, S. The four dimensions of contestable ai diagnostics-a patient-centric approach to explainable ai. *Artificial Intelligence in Medicine* **107**, 101901 (2020).

[12] Amann, J., Blasimme, A., Vayena, E., Frey, D. & Madai, V. I. Explainability for artificial intelligence in healthcare: a multidisciplinary perspective. *BMC Medical Informatics and Decision Making* **20**, 1–9 (2020).

[13] Norman, D. A. Affordance, conventions, and design. *Interactions* **6**, 38–43 (1999).

[14] Cai, C. J. *et al.* Human-centered tools for coping with imperfect algorithms during medical decision-making. In *Proceedings of the 2019 CHI Conference on Human Factors in Computing Systems*, 1–14 (2019).

[15] Xie, Y., Chen, M., Kao, D., Gao, G. & Chen, X. Chexplain: Enabling physicians to explore and understand data-driven, AI-enabled medical imaging analysis. In *Proceedings of the 2020 CHI Conference on Human Factors in Computing Systems*, 1–13 (2020).

[16] Jacobs, M. *et al.* Designing AI for trust and collaboration in time-constrained medical decisions: A sociotechnical lens. In *Proceedings of the 2021 CHI Conference on Human Factors in Computing Systems*, 1–14 (2021).

[17] Suresh, H., Gomez, S. R., Nam, K. K. & Satyanarayan, A. Beyond expertise and roles: A framework to characterize the stakeholders of interpretable machine learning and their needs. In *Proceedings of the 2021 CHI Conference on Human Factors in Computing Systems*, 1–16 (2021).

[18] Lai, V. & Tan, C. On human predictions with explanations and predictions of machine learning models: A case study on deception detection. In *Proceedings of the conference on fairness, accountability, and transparency*, 29–38 (2019).

[19] Eiband, M. *et al.* Bringing transparency design into practice. In *23rd international conference on intelligent user interfaces*, 211–223 (2018).

[20] Wang, X. & Yin, M. Are explanations helpful? a comparative study of the effects of explanations in AI-assisted decision-making. In *26th International Conference on Intelligent User Interfaces*, 318–328 (2021).

[21] Cheng, H.-F. *et al.* Explaining decision-making algorithms through ui: Strategies to help non-expert stakeholders. In *Proceedings of the 2019 chi conference on human factors in computing systems*, 1–12 (2019).

[22] Smith-Renner, A. *et al.* No explainability without accountability: An empirical study of explanations and feedback in interactive ml. In *Proceedings of the 2020 CHI Conference on Human Factors in Computing Systems*, 1–13 (2020).

[23] Bansal, G. *et al.* Does the whole exceed its parts? the effect of ai explanations on complementary team performance. In *Proceedings of the 2021 CHI Conference on Human Factors in Computing Systems*, 1–16 (2021).

[24] Bansal, G. *et al.* Beyond accuracy: The role of mental models in human-AI team performance. In *Proceedings of the AAAI Conference on Human Computation and Crowdsourcing*, vol. 7, 2–11 (2019).

[25] Nourani, M. *et al.* Anchoring bias affects mental model formation and user reliance in explainable ai systems. In *26th International Conference on Intelligent User Interfaces*, 340–350 (2021).

[26] McCoy, L. G., Brenna, C. T., Chen, S., Vold, K. & Das, S. Believing in black boxes: Machine learning for healthcare does not need explainability to be evidence-based. *Journal of Clinical Epidemiology* (2021).

[27] Deeley, M. *et al.* Segmentation editing improves efficiency while reducing inter-expert variation and maintaining accuracy for normal brain tissues in the presence of space-occupying lesions. *Physics in Medicine & Biology* **58**, 4071 (2013).

[28] Banerjee, I. *et al.* Reading race: Ai recognises patient's racial identity in medical images. *preprint at https://arxiv.org/abs/2107.10356* (2021).

[29] Liu, T. A. *et al.* Gene expression profile prediction in uveal melanoma using deep learning: A pilot study for the development of an alternative survival prediction tool. *Ophthalmology Retina* **4**, 1213–1215 (2020).

[30] Lu, M. Y. *et al.* Deep learning-based computational pathology predicts origins for cancers of unknown primary. *preprint at https://arxiv.org/abs/2006.13932* (2020).

[31] Cai, C. J., Winter, S., Steiner, D., Wilcox, L. & Terry, M. " hello AI": Uncovering the onboarding needs of medical practitioners for human-ai collaborative decision-making. *Proceedings of the ACM on Human-computer Interaction* **3**, 1–24 (2019).

[32] Wang, D., Yang, Q., Abdul, A. & Lim, B. Y. Designing theory-driven user-centric explainable AI. In *Proceedings of the 2019 CHI conference on human factors in computing systems*, 1–15 (2019).

[33] Nourani, M., King, J. & Ragan, E. The role of domain expertise in user trust and the impact of first impressions with intelligent systems. In *Proceedings of the AAAI Conference on Human Computation and Crowdsourcing*, vol. 8, 112–121 (2020).

[34] Buçinca, Z., Malaya, M. B. & Gajos, K. Z. To trust or to think: Cognitive forcing functions can reduce overreliance on ai in ai-assisted decision-making. *Proceedings of the ACM on Human-Computer Interaction* **5**, 1–21 (2021).

[35] Gaube, S. *et al.* Do as ai say: susceptibility in deployment of clinical decision-aids. *NPJ Digital Medicine* **4**, 1–8 (2021).

[36] Amershi, S. *et al.* Guidelines for human-AI interaction. In *Proceedings of the 2019 chi conference on human factors in computing systems*, 1–13 (2019).

[37] Liao, Q. V., Gruen, D. & Miller, S. Questioning the AI: informing design practices for explainable ai user experiences. In *Proceedings of the 2020 CHI Conference on Human Factors in Computing Systems*, 1–15 (2020).

[38] Mohseni, S., Zarei, N. & Ragan, E. D. A multidisciplinary survey and framework for design and evaluation of explainable AI systems. *ACM Transactions on Interactive Intelligent Systems (TiiS)* **11**, 1–45 (2021).

[39] Chen, J., Kallus, N., Mao, X., Svacha, G. & Udell, M. Fairness under unawareness: Assessing disparity when protected class is unobserved. In *Proceedings of the conference on fairness, accountability, and transparency*, 339–348 (2019).

[40] Datta, A., Tschantz, M. C. & Datta, A. Automated experiments on ad privacy settings: A tale of opacity, choice, and discrimination. *preprint at https://arxiv.org/abs/1408.6491* (2014).

[41] Leslie, D. Understanding artificial intelligence ethics and safety: A guide for the responsible design and implementation of ai systems in the public sector. *Available at SSRN 3403301* (2019).

[42] Sokol, K. & Flach, P. Explainability fact sheets: a framework for systematic assessment of explainable approaches. In *Proceedings of the 2020 Conference on Fairness, Accountability, and Transparency*, 56–67 (2020).

[43] Liu, X., Rivera, S. C., Moher, D., Calvert, M. J. & Denniston, A. K. Reporting guidelines for clinical trial reports for interventions involving artificial intelligence: the consort-AI extension. *BMJ* **370** (2020).

[44] Decide-ai: new reporting guidelines to bridge the development-to-implementation gap in clinical artificial intelligence. *Nature Medicine* **27**, 186–187 (2021).

[45] Cabitza, F. & Campagner, A. The need to separate the wheat from the chaff in medical informatics: Introducing a comprehensive checklist for the (self)-assessment of medical ai studies (2021).

[46] Hernandez-Boussard, T., Bozkurt, S., Ioannidis, J. P. & Shah, N. H. Minimar (minimum information for medical ai reporting): developing reporting standards for artificial intelligence in health care. *Journal of the American Medical Informatics Association* **27**, 2011–2015 (2020).

[47] Scott, I., Carter, S. & Coiera, E. Clinician checklist for assessing suitability of machine learning applications in healthcare. *BMJ Health & Care Informatics* **28** (2021).

[48] Tjoa, E. & Guan, C. A survey on explainable artificial intelligence (xai): Toward medical xai. *IEEE transactions on neural networks and learning systems* **32**, 4793–4813 (2020).

[49] van der Velden, B. H., Kuijf, H. J., Gilhuijs, K. G. & Viergever, M. A. Explainable artificial intelligence (xai) in deep learning-based medical image analysis. *Medical Image Analysis* 102470 (2022).

[50] Gulum, M. A., Trombley, C. M. & Kantardzic, M. A review of explainable deep learning cancer detection models in medical imaging. *Applied Sciences* **11**, 4573 (2021).

[51] Krizhevsky, A., Sutskever, I. & Hinton, G. E. Imagenet classification with deep convolutional neural networks. *Communications of the ACM* **60**, 84–90 (2017).

[52] Moher, D., Liberati, A., Tetzlaff, J., Altman, D. G. & Group, P. Preferred reporting items for systematic reviews and meta-analyses: the prisma statement. *PLoS Medicine* **6**, e1000097 (2009).

[53] Rudin, C. Stop explaining black box machine learning models for high stakes decisions and use interpretable models instead. *Nature Machine Intelligence* **1**, 206–215 (2019).

[54] Molnar, C. *Interpretable machine learning* (Lulu. com, 2020).

[55] Abdel Magid, S. *et al.* Channel Embedding for Informative Protein Identification from Highly Multiplexed Images. *23rd International Conference on Medical Image Computing and Computer-Assisted Intervention, MICCAI 2020, October 4, 2020 - October 8, 2020* **12265 LNCS**, 3–13 (2020). URL `http://dx.doi.org/10.1007/978-3-030-59722-1_1NS-`.

[56] Afshar, P. *et al.* MIXCAPS: A capsule network-based mixture of experts for lung nodule malignancy prediction. *Pattern Recognition* **116** (2021). URL `http://dx.doi.org/10.1016/j.patcog.2021.107942NS-`.

[57] Fan, M., Chakraborti, T., Chang, E. I. C., Xu, Y. & Rittscher, J. Microscopic Fine-Grained Instance Classification Through Deep Attention. *23rd International Conference on Medical Image Computing and Computer-Assisted Intervention, MICCAI 2020, October 4, 2020 - October 8, 2020* **12265 LNCS**, 490–499 (2020). URL `http://dx.doi.org/10.1007/978-3-030-59722-1_47NS-`.

[58] Graziani, M., Lompech, T., Muller, H., Depeursinge, A. & Andrearczyk, V. Interpretable CNN Pruning for Preserving Scale-Covariant Features in Medical Imaging. *3rd International Workshop on Interpretability of Machine Intelligence in Medical Image Computing, iMIMIC 2020, the 2nd International Workshop on Medical Image Learning with Less Labels and Imperfect Data, MIL3ID 2020, and the 5th International Workshop o* **12446 LNCS**, 23–32 (2020). URL `http://dx.doi.org/10.1007/978-3-030-61166-8_3NS-`.

[59] An, F., Li, X. & Ma, X. Medical Image Classification Algorithm Based on Visual Attention Mechanism-MCNN. *Oxidative Medicine and Cellular Longevity* **2021** (2021). URL https://www.embase.com/search/results?subaction=viewrecord&id=L2011217895&from=exporthttp://dx.doi.org/10.1155/2021/6280690.

[60] He, S. *et al.* Multi-channel attention-fusion neural network for brain age estimation: Accuracy, generality, and interpretation with 16,705 healthy MRIs across lifespan. *Medical Image Analysis* **72** (2021). URL https://www.embase.com/search/results?subaction=viewrecord&id=L2012117928&from=exporthttp://dx.doi.org/10.1016/j.media.2021.102091.

[61] Hou, B., Kang, G., Xu, X. & Hu, C. Cross Attention Densely Connected Networks for Multiple Sclerosis Lesion Segmentation. *2019 IEEE International Conference on Bioinformatics and Biomedicine, BIBM 2019, November 18, 2019 - November 21, 2019* 2356–2361 (2019). URL http://dx.doi.org/10.1109/BIBM47256.2019.8983149NS-.

[62] Huang, Y. & Chung, A. C. S. Evidence localization for pathology images using weakly supervised learning. *22nd International Conference on Medical Image Computing and Computer-Assisted Intervention, MICCAI 2019, October 13, 2019 - October 17, 2019* **11764 LNCS**, 613–621 (2019). URL http://dx.doi.org/10.1007/978-3-030-32239-7_68NS-.

[63] Morvan, L. *et al.* Learned Deep Radiomics for Survival Analysis with Attention. *3rd International Workshop on Predictive Intelligence in Medicine, PRIME 2020, held in conjunction with the Medical Image Computing and Computer Assisted Intervention, MICCAI 2020, October 8, 2020 - October 8, 2020* **12329 LNCS**, 35–45 (2020). URL http://dx.doi.org/10.1007/978-3-030-59354-4_4NS-.

[64] Saleem, H., Shahid, A. R. & Raza, B. Visual interpretability in 3D brain tumor segmentation network. *Computers in Biology and Medicine* **133** (2021). URL https://www.embase.com/search/results?subaction=viewrecord&id=L2011734982&from=exporthttp://dx.doi.org/10.1016/j.compbiomed.2021.104410.

[65] Shahamat, H. & Saniee Abadeh, M. Brain MRI analysis using a deep learning based evolutionary approach. *Neural Networks* **126**, 218–234 (2020). URL https://www.embase.com/search/results?subaction=viewrecord&id=L2005472077&from=exporthttp://dx.doi.org/10.1016/j.neunet.2020.03.017.

[66] Singla, S. *et al.* Subject2Vec: Generative-Discriminative Approach from a Set of Image Patches to a Vector. *Med Image Comput Comput Assist Interv* **11070**, 502–510 (2018). URL NS-.

[67] Sun, J., Darbehani, F., Zaidi, M. & Wang, B. SAUNet: Shape Attentive U-Net for Interpretable Medical Image Segmentation. *23rd International Conference on Medical Image Computing and Computer-Assisted Intervention, MICCAI 2020, October 4, 2020 - October 8, 2020* **12264 LNCS**, 797–806 (2020). URL http://dx.doi.org/10.1007/978-3-030-59719-1_77NS-.

[68] Xu, X. *et al.* Automatic glaucoma detection based on transfer induced attention network. *Biomedical Engineering Online* **20**, 39 (2021). URL https://www.embase.com/search/results?subaction=viewrecord&id=L634874614&from=exporthttp://dx.doi.org/10.1186/s12938-021-00877-5.

[69] Yang, H., Kim, J.-Y., Kim, H. & Adhikari, S. P. Guided Soft Attention Network for Classification of Breast Cancer Histopathology Images. *IEEE Transactions on Medical Imaging* **39**, 1306–1315 (2020). URL http://dx.doi.org/10.1109/TMI.2019.2948026NS-.

[70] Diao, J. A. *et al.* Human-interpretable image features derived from densely mapped cancer pathology slides predict diverse molecular phenotypes. *Nature Communications* **12** (2021). URL https://www.embase.com/search/results?subaction=viewrecord&id=L2010776995&from=exporthttp://dx.doi.org/10.1038/s41467-021-21896-9.

[71] Dong, Y. *et al.* A Polarization-Imaging-Based Machine Learning Framework for Quantitative Pathological Diagnosis of Cervical Precancerous Lesions. *IEEE Transactions on Medical Imaging* (2021). URL https://www.embase.com/search/results?subaction=viewrecord&id=L635538309&from=exporthttp://dx.doi.org/10.1109/TMI.2021.3097200.

[72] Giannini, V., Rosati, S., Regge, D. & Balestra, G. Texture features and artificial neural networks: A way to improve the specificity of a CAD system for multiparametric MR prostate cancer. *14th Mediterranean Conference on Medical and Biological Engineering and Computing, MEDICON 2016, March 31, 2016 - April 2, 2016* **57**, 296–301 (2016). URL http://dx.doi.org/10.1007/978-3-319-32703-7_59NS-.

[73] Loveymi, S., Dezfoulian, M. H. & Mansoorizadeh, M. Generate Structured Radiology Report from CT Images Using Image Annotation Techniques: Preliminary Results with Liver CT. *Journal of Digital Imaging* **33**, 375–390 (2020). URL http://dx.doi.org/10.1007/s10278-019-00298-wNS-.

[74] MacCormick, I. J. C. *et al.* Accurate, fast, data efficient and interpretable glaucoma diagnosis with automated spatial analysis of the whole cup to disc profile. *PLoS ONE* **14** (2019). URL https://www.embase.com/search/results?subaction=viewrecord&id=L625837308&from=exporthttp://dx.doi.org/10.1371/journal.pone.0209409.

[75] Kunapuli, G. *et al.* A Decision-Support Tool for Renal Mass Classification. *Journal of Digital Imaging* **31**, 929–939 (2018). URL https://www.embase.com/search/results?subaction=viewrecord&id=L625181034&from=exporthttp://dx.doi.org/10.1007/s10278-018-0100-0.

[76] Shen, T., Wang, J., Gou, C. & Wang, F.-Y. Hierarchical Fused Model with Deep Learning and Type-2 Fuzzy Learning for Breast Cancer Diagnosis. *IEEE Transactions on Fuzzy Systems* **28**, 3204–3218 (2020). URL http://dx.doi.org/10.1109/TFUZZ.2020.3013681NS-.

[77] Li, J., Shi, H. & Hwang, K.-S. An explainable ensemble feedforward method with Gaussian convolutional filter. *Knowledge-Based Systems* **225** (2021). URL http://dx.doi.org/10.1016/j.knosys.2021.107103NS-.

[78] Puyol-Anton, E. *et al.* Assessing the Impact of Blood Pressure on Cardiac Function Using Interpretable Biomarkers and Variational Autoencoders. *10th International Workshop on Statistical Atlases and Computational Models of the Heart, STACOM 2019, held in conjunction with the 22nd International Conference on Medical Image Computing and Computer Assisted Intervention, MICCAI 2019, October 13, 2019* **12009 LNCS**, 22–30 (2020). URL http://dx.doi.org/10.1007/978-3-030-39074-7_3NS-.

[79] Wongvibulsin, S., Wu, K. C. & Zeger, S. L. Improving Clinical Translation of Machine Learning Approaches Through Clinician-Tailored Visual Displays of Black Box Algorithms: Development and Validation. *JMIR Med Inform* **8**, e15791 (2020). URL NS-.

[80] Lin, Y., Wei, L., Han, S. X., Aberle, D. R. & Hsu, W. EDICNet: An end-to-end detection and interpretable malignancy classification network for pulmonary nodules in computed tomography. *Medical Imaging 2020: Computer-Aided Diagnosis, February 16, 2020 - February 19, 2020* **11314**, The Society of Photo–Optical Instrumentation Engin (2020). URL http://dx.doi.org/10.1117/12.2551220NS-.

[81] Kim, S. T., Lee, H., Kim, H. G. & Ro, Y. M. ICADx: Interpretable computer aided diagnosis of breast masses. *Medical Imaging 2018: Computer-Aided Diagnosis, February 12, 2018 - February 15, 2018* **10575**, DECTRIS Ltd.; The Society of Photo–Optical Instrum (2018). URL http://dx.doi.org/10.1117/12.2293570NS-.

[82] Kim, S. T., Lee, J.-H., Lee, H. & Ro, Y. M. Visually interpretable deep network for diagnosis of breast masses on mammograms. *Physics in Medicine and Biology* **63**, 235025 (2018). URL https://www.embase.com/search/results?subaction=viewrecord&id=L628170713&from=exporthttp://dx.doi.org/10.1088/1361-6560/aaef0a.

[83] Puyol-Antón, E. *et al.* Interpretable Deep Models for Cardiac Resynchronisation Therapy Response Prediction. *Med Image Comput Comput Assist Interv* **2020**, 284–293 (2020). URL NS-.

[84] Wang, C. J. *et al.* Deep learning for liver tumor diagnosis part II: convolutional neural network interpretation using radiologic imaging features. *European Radiology* **29**, 3348–3357 (2019). URL https://www.embase.com/search/results?subaction=viewrecord&id=L627809141&from=exporthttp://dx.doi.org/10.1007/s00330-019-06214-8.

[85] Codella, N. C. F. *et al.* Collaborative human-AI (CHAI): Evidence-based interpretable melanoma classification in dermoscopic images. *1st International Workshop on Machine Learning in Clinical Neuroimaging, MLCN 2018, 1st International Workshop on Deep Learning Fails, DLF 2018, and 1st International Workshop on Interpretability of Machine Intelligence in Medical Image Computing, iMIMIC* **11038 LNCS**, 97–105 (2018). URL http://dx.doi.org/10.1007/978-3-030-02628-8_11NS-.

[86] Barata, C., Celebi, M. E. & Marques, J. S. Explainable skin lesion diagnosis using taxonomies. *Pattern Recognition* **110** (2021). URL http://dx.doi.org/10.1016/j.patcog.2020.107413NS-.

[87] Silva, W., Fernandes, K., Cardoso, M. J. & Cardoso, J. S. Towards complementary explanations using deep neural networks. *1st International Workshop on Machine Learning in Clinical Neuroimaging, MLCN 2018, 1st International Workshop on Deep Learning Fails, DLF 2018, and 1st International Workshop on Interpretability of Machine Intelligence in Medical Image Computing, iMIMIC* **11038 LNCS**, 133–140 (2018). URL http://dx.doi.org/10.1007/978-3-030-02628-8_15NS-.

[88] Khaleel, M., Tavanapong, W., Wong, J., Oh, J. & De Groen, P. Hierarchical visual concept interpretation for medical image classification. *34th IEEE International Symposium on Computer-Based Medical Systems, CBMS 2021, June 7, 2021 - June 9, 2021* **2021-June**, 25–30 (2021). URL http://dx.doi.org/10.1109/CBMS52027.2021.00012NS-.

[89] Pereira, S. *et al.* Enhancing interpretability of automatically extracted machine learning features: application to a RBM-Random Forest system on brain lesion segmentation. *Medical Image Analysis* **44**, 228–244 (2018). URL https://www.embase.com/search/results?subaction=viewrecord&id=L619966103&from=exporthttp://dx.doi.org/10.1016/j.media.2017.12.009.

[90] Yan, K. *et al.* Holistic and comprehensive annotation of clinically significant findings on diverse CT images: Learning from radiology reports and label ontology. *32nd IEEE/CVF Conference on Computer Vision and Pattern Recognition, CVPR 2019, June 16, 2019 - June 20, 2019* **2019-June**, 8515–8524 (2019). URL http://dx.doi.org/10.1109/CVPR.2019.00872NS-.

[91] Chen, H., Miao, S., Xu, D., Hager, G. D. & Harrison, A. P. Deep hiearchical multi-label classification applied to chest x-ray abnormality taxonomies. *Medical Image Analysis* **66**, 101811 (2020).

[92] Verma, A., Shukla, P., Abhishek & Verma, S. An interpretable SVM based model for cancer prediction in mammograms. *1st International Conference on Communication, Networks and Computing, CNC 2018, March 22, 2018 - March 24, 2018* **839**, 443–451 (2019). URL http://dx.doi.org/10.1007/978-981-13-2372-0_39NS-.

[93] Li, Y. *et al.* Computer-Aided Cervical Cancer Diagnosis Using Time-Lapsed Colposcopic Images. *IEEE Transactions on Medical Imaging* **39**, 3403–3415 (2020). URL https://www.embase.com/search/results?subaction=viewrecord&id=L631763414&from=exporthttp://dx.doi.org/10.1109/TMI.2020.2994778.

[94] Wang, K. *et al.* A dual-mode deep transfer learning (D2TL) system for breast cancer detection using contrast enhanced digital mammograms. *IISE Transactions on Healthcare Systems Engineering* **9**, 357–370 (2019). URL https://www.embase.com/search/results?subaction=viewrecord&id=L628355909&from=exporthttp://dx.doi.org/10.1080/24725579.2019.1628133.

[95] Zhao, G., Zhou, B., Wang, K., Jiang, R. & Xu, M. Respond-CAM: Analyzing deep models for 3D imaging data by visualizations. *21st International Conference on Medical Image Computing and Computer Assisted Intervention, MICCAI 2018, September 16, 2018 - September 20, 2018* **11070 LNCS**, 485–492 (2018). URL http://dx.doi.org/10.1007/978-3-030-00928-1_55NS-.

[96] Folke, T., Yang, S. C.-H., Anderson, S. & Shafto, P. Explainable AI for medical imaging: Explaining pneumothorax diagnoses with Bayesian teaching. *Artificial Intelligence and Machine Learning for Multi-Domain Operations Applications III 2021, April 12, 2021 - April 16, 2021* **11746**, The Society of Photo–Optical Instrumentation Engin (2021). URL http://dx.doi.org/10.1117/12.2585967NS-.

[97] Liao, W. *et al.* Clinical Interpretable Deep Learning Model for Glaucoma Diagnosis. *IEEE Journal of Biomedical and Health Informatics* **24**, 1405–1412 (2020). URL https://www.embase.com/search/results?subaction=viewrecord&id=L631748506&from=exporthttp://dx.doi.org/10.1109/JBHI.2019.2949075.

[98] Shinde, S., Chougule, T., Saini, J. & Ingalhalikar, M. HR-CAM: Precise localization of pathology using multi-level learning in CNNS. *22nd International Conference on Medical Image Computing and Computer-Assisted Intervention, MICCAI 2019, October 13, 2019 - October 17, 2019* **11767 LNCS**, 298–306 (2019). URL http://dx.doi.org/10.1007/978-3-030-32251-9_33NS-.

[99] Ballard, D. H. Modular learning in neural networks. In *AAAI*, vol. 647, 279–284 (1987).

[100] Biffi, C. *et al.* Learning interpretable anatomical features through deep generative models: Application to cardiac remodeling. *21st International Conference on Medical Image Computing and Computer Assisted Intervention, MICCAI 2018, September 16, 2018 - September 20, 2018* **11071 LNCS**, 464–471 (2018). URL http://dx.doi.org/10.1007/978-3-030-00934-2_52NS-.

[101] Couteaux, V., Nempont, O., Pizaine, G. & Bloch, I. Towards interpretability of segmentation networks by analyzing deepDreams. *2nd International Workshop on Interpretability of Machine Intelligence in Medical Image Computing, iMIMIC 2019, and the 9th International Workshop on Multimodal Learning for Clinical Decision Support, ML-CDS 2019, held in conjunction with the 22nd Interna* **11797 LNCS**, 56–63 (2019). URL http://dx.doi.org/10.1007/978-3-030-33850-3_7NS-.

[102] Guo, X. *et al.* Intelligent medical image grouping through interactive learning. *International Journal of Data Science and Analytics* **2**, 95–105 (2016). URL http://dx.doi.org/10.1007/s41060-016-0021-2NS-.

[103] Janik, A., Dodd, J., Ifrim, G., Sankaran, K. & Curran, K. Interpretability of a deep learning model in the application of cardiac MRI segmentation with an ACDC challenge dataset. *Medical Imaging 2021: Image Processing, February 15, 2021 - February 19, 2021* **11596**, The Society of Photo–Optical Instrumentation Engin (2021). URL `http://dx.doi.org/10.1117/12.2582227NS-`.

[104] Sari, C. T. & Gunduz-Demir, C. Unsupervised Feature Extraction via Deep Learning for Histopathological Classification of Colon Tissue Images. *IEEE Transactions on Medical Imaging* **38**, 1139–1149 (2019). URL `http://dx.doi.org/10.1109/TMI.2018.2879369NS-`.

[105] Venugopalan, J., Tong, L., Hassanzadeh, H. R. & Wang, M. D. Multimodal deep learning models for early detection of Alzheimer's disease stage. *Scientific Reports* **11**, 3254 (2021). URL `https://www.embase.com/search/results?subaction=viewrecord&id=L634212207&from=exporthttp://dx.doi.org/10.1038/s41598-020-74399-w`.

[106] Zhu, P. & Ogino, M. Guideline-based additive explanation for computer-aided diagnosis of lung nodules. *2nd International Workshop on Interpretability of Machine Intelligence in Medical Image Computing, iMIMIC 2019, and the 9th International Workshop on Multimodal Learning for Clinical Decision Support, ML-CDS 2019, held in conjunction with the 22nd Interna* **11797 LNCS**, 39–47 (2019). URL `http://dx.doi.org/10.1007/978-3-030-33850-3_5NS-`.

[107] Pirovano, A., Heuberger, H., Berlemont, S., Ladjal, S. & Bloch, I. Improving Interpretability for Computer-Aided Diagnosis Tools on Whole Slide Imaging with Multiple Instance Learning and Gradient-Based Explanations. *3rd International Workshop on Interpretability of Machine Intelligence in Medical Image Computing, iMIMIC 2020, the 2nd International Workshop on Medical Image Learning with Less Labels and Imperfect Data, MIL3ID 2020, and the 5th International Workshop o* **12446 LNCS**, 43–53 (2020). URL `http://dx.doi.org/10.1007/978-3-030-61166-8_5NS-`.

[108] Hao, J., Kosaraju, S. C., Tsaku, N. Z., Song, D. H. & Kang, M. PAGE-Net: Interpretable and Integrative Deep Learning for Survival Analysis Using Histopathological Images and Genomic Data. *Pacific Symposium on Biocomputing. Pacific Symposium on Biocomputing* **25**, 355–366 (2020). URL `https://www.embase.com/search/results?subaction=viewrecord&id=L630059597&from=export`.

[109] de Sousa, I. P., Vellasco, M. M. B. R. & da Silva, E. C. Approximate Explanations for Classification of Histopathology Patches. *Workshops of the 20th Joint European Conference on Machine Learning and Knowledge Discovery in Databases, ECML PKDD, September 14, 2020 - September 18, 2020* **1323**, 517–526 (2020). URL `http://dx.doi.org/10.1007/978-3-030-65965-3_35NS-`.

[110] Li, X., Dvornek, N. C., Zhuang, J., Ventola, P. & Duncan, J. S. Brain biomarker interpretation in ASD using deep learning and fMRI. *21st International Conference on Medical Image Computing and Computer Assisted Intervention, MICCAI 2018, September 16, 2018 - September 20, 2018* **11072 LNCS**, 206–214 (2018). URL `http://dx.doi.org/10.1007/978-3-030-00931-1_24NS-`.

[111] Quellec, G. *et al.* ExplAIn: Explanatory artificial intelligence for diabetic retinopathy diagnosis. *Medical Image Analysis* **72** (2021). URL `https://www.embase.com/search/results?subaction=viewrecord&id=L2012995582&from=exporthttp://dx.doi.org/10.1016/j.media.2021.102118`.

[112] Uzunova, H., Ehrhardt, J., Kepp, T. & Handels, H. Interpretable explanations of black box classifiers applied on medical images by meaningful perturbations using variational autoencoders. *Medical Imaging 2019: Image Processing, February 19, 2019 - February 21, 2019* **10949**, The Society of Photo–Optical Instrumentation Engin (2019). URL `http://dx.doi.org/10.1117/12.2511964NS-`.

[113] Liu, J. *et al.* Ultrasound Liver Fibrosis Diagnosis Using Multi-indicator Guided Deep Neural Networks. *10th International Workshop on Machine Learning in Medical Imaging, MLMI 2019 held in conjunction with the 22nd International Conference on Medical Image Computing and Computer-Assisted Intervention, MICCAI 2019, October 13, 2019 - October 13, 2019* **11861 LNCS**, 230–237 (2019). URL `http://dx.doi.org/10.1007/978-3-030-32692-0_27NS-`.

[114] Liu, Y. *et al.* Act Like a Radiologist: Towards Reliable Multi-view Correspondence Reasoning for Mammogram Mass Detection. *IEEE Transactions on Pattern Analysis and Machine Intelligence* (2021). URL `http://dx.doi.org/10.1109/TPAMI.2021.3085783NS-`.

[115] Oktay, O. *et al.* Anatomically Constrained Neural Networks (ACNNs): Application to Cardiac Image Enhancement and Segmentation. *IEEE Transactions on Medical Imaging* **37**, 384–395 (2018). URL `http://dx.doi.org/10.1109/TMI.2017.2743464NS-`.

[116] Peng, T., Boxberg, M., Weichert, W., Navab, N. & Marr, C. Multi-task learning of a deep K-nearest neighbour network for histopathological image classification and retrieval. *22nd International Conference on Medical Image Computing and Computer-Assisted Intervention, MICCAI 2019, October 13, 2019 - October 17, 2019* **11764 LNCS**, 676–684 (2019). URL http://dx.doi.org/10.1007/978-3-030-32239-7_75NS-.

[117] Liu, Y., Li, Z., Ge, Q., Lin, N. & Xiong, M. Deep Feature Selection and Causal Analysis of Alzheimer's Disease. *Frontiers in Neuroscience* **13** (2019). URL https://www.embase.com/search/results?subaction=viewrecord&id=L629992085&from=exporthttp://dx.doi.org/10.3389/fnins.2019.01198.

[118] Ren, H. *et al.* Interpretable Pneumonia Detection by Combining Deep Learning and Explainable Models with Multisource Data. *Ieee Access* **9**, 95872–95883 (2021). URL http://dx.doi.org/10.1109/ACCESS.2021.3090215NS-.

[119] Velikova, M., Lucas, P. J. F., Samulski, M. & Karssemeijer, N. On the interplay of machine learning and background knowledge in image interpretation by Bayesian networks. *Artificial Intelligence in Medicine* **57**, 73–86 (2013). URL https://www.embase.com/search/results?subaction=viewrecord&id=L52426996&from=exporthttp://dx.doi.org/10.1016/j.artmed.2012.12.004.

[120] Carneiro, G., Zorron Cheng Tao Pu, L., Singh, R. & Burt, A. Deep learning uncertainty and confidence calibration for the five-class polyp classification from colonoscopy. *Medical Image Analysis* **62** (2020). URL https://www.embase.com/search/results?subaction=viewrecord&id=L2005189093&from=exporthttp://dx.doi.org/10.1016/j.media.2020.101653.

[121] Sabol, P. *et al.* Explainable classifier for improving the accountability in decision-making for colorectal cancer diagnosis from histopathological images. *Journal of Biomedical Informatics* **109** (2020). URL https://www.embase.com/search/results?subaction=viewrecord&id=L2007460563&from=exporthttp://dx.doi.org/10.1016/j.jbi.2020.103523.

[122] Tanno, R. *et al.* Uncertainty modelling in deep learning for safer neuroimage enhancement: Demonstration in diffusion MRI. *NeuroImage* **225** (2021). URL https://www.embase.com/search/results?subaction=viewrecord&id=L2008373754&from=exporthttp://dx.doi.org/10.1016/j.neuroimage.2020.117366.

[123] Doshi-Velez, F. & Kim, B. Towards a rigorous science of interpretable machine learning. *preprint at https://arxiv.org/abs/1702.08608* (2017).

[124] Adebayo, J. *et al.* Sanity checks for saliency maps. In Bengio, S. *et al.* (eds.) *Advances in Neural Information Processing Systems*, vol. 31 (Curran Associates, Inc., 2018). URL https://proceedings.neurips.cc/paper/2018/file/294a8ed24b1ad22ec2e7efea049b8737-Paper.pdf.

[125] Yeche, H., Harrison, J. & Berthier, T. UBS: A dimension-agnostic metric for concept vector interpretability applied to radiomics. *2nd International Workshop on Interpretability of Machine Intelligence in Medical Image Computing, iMIMIC 2019, and the 9th International Workshop on Multimodal Learning for Clinical Decision Support, ML-CDS 2019, held in conjunction with the 22nd Interna* **11797 LNCS**, 12–20 (2019). URL http://dx.doi.org/10.1007/978-3-030-33850-3_2NS-.

[126] Chen, H., Miao, S., Xu, D., Hager, G. D. & Harrison, A. P. Deep hierarchical multi-label classification of chest x-ray images. In *International Conference on Medical Imaging with Deep Learning*, 109–120 (PMLR, 2019).

[127] Zhang, Z. *et al.* Origa-light: An online retinal fundus image database for glaucoma analysis and research. In *2010 Annual International Conference of the IEEE Engineering in Medicine and Biology*, 3065–3068 (IEEE, 2010).

[128] Menze, B. H. *et al.* The multimodal brain tumor image segmentation benchmark (brats). *IEEE Transactions on Medical Imaging* **34**, 1993–2024 (2014).
